# Medicinal Uses, Phytochemistry, Pharmacology, and Toxicology of *Mentha spicata*

**DOI:** 10.1155/2022/7990508

**Published:** 2022-04-12

**Authors:** Naoual El Menyiy, Hanae Naceiri Mrabti, Nasreddine El Omari, Afaf EI Bakili, Saad Bakrim, Mouna Mekkaoui, Abdelaali Balahbib, Ehsan Amiri-Ardekani, Riaz Ullah, Ali S. Alqahtani, Abdelaaty A. Shahat, Abdelhakim Bouyahya

**Affiliations:** ^1^Laboratory of Pharmacology, National Agency of Medicinal and Aromatic Plants, Taounate 34025, Morocco; ^2^Laboratory of Pharmacology and Toxicology, Bio Pharmaceutical and Toxicological Analysis Research Team, Faculty of Medicine and Pharmacy, Mohammed V University, BP 6203, Rabat, Morocco; ^3^Laboratory of Histology, Embryology and Cytogenetic, Faculty of Medicine and Pharmacy, Mohammed V University, Rabat, Morocco; ^4^Mohammed V University in Rabat, LPCMIO, Materials Science Center (MSC), Ecole Normale Supérieure, Rabat, Morocco; ^5^Molecular Engineering, Valorization and Environment Team, Department of Science and Technology, Polydisciplinary Faculty of Taroudant, Ibn Zohr University, Taroudant, Morocco; ^6^Pharmacodynamics Research Team, Laboratory of Pharmacology and Toxicology, Faculty of Medicine and Pharmacy, University Mohammed V in Rabat, Morocco; ^7^Laboratory of Biodiversity, Ecology and Genome, Faculty of Sciences, Mohammed V University, Rabat 10106, Morocco; ^8^Department of Phytopharmaceuticals (Traditional Pharmacy), Faculty of Pharmacy, Shiraz University of Medical Sciences, Shiraz, Iran; ^9^Department of Pharmacognosy (Medicinal Aromatic and Posnous Plants Research Center, College of Pharmacy, King Saud University, Riyadh, Saudi Arabia; ^10^Laboratory of Human Pathologies Biology, Department of Biology, Faculty of Sciences and Genomic Center of Human Pathologies, Faculty of Medicine and Pharmacy, Mohammed V University in Rabat, Rabat, Morocco

## Abstract

*Mentha spicata*, also called *Mentha viridis*, is a medicinal plant of the Lamiaceae family characterized by its potency to synthesize and secret secondary metabolites, essentially essential oils. Different populations use the aerial parts of this plant for tea preparation, and this tisane has shown several effects, according to ethnopharmacological surveys carried out in different areas around the world. These effects are attributed to different compounds of *M. spicata*, in which their biological effects were recently proved experimentally. Pharmacological properties of *M. spicata* extracts and essential oils were investigated for different health benefits such as antioxidant, anticancer, antiparasitic, antimicrobial, and antidiabetic effects. In vitro and in vivo studies showed positives effects that could be certainly related to different bioactive compounds identified in *M. spicata*. Indeed, volatile compounds seem to be efficient in inhibiting different microbial agents such as bacteria, fungi, and parasites through several mechanisms. Moreover, *M. spicata* exhibited, according to some studies, promising antioxidant, antidiabetic, anti-inflammatory, and anticancer effects, which show its potential to be used as a source for identifying natural drugs against cellular oxidative stress and its related diseases. Importantly, toxicological investigations of *M. spicata* show the safety of this species at different doses and several periods of use which justify its use in traditional medicines as tisane with tea. Here, we report, explore, and highlight the data published on *M. spicata* concerning its botanical description and geographical distribution, its phytochemical compounds, its pharmacological properties, and its toxicological investigations of *M. spicata*.

## 1. Introduction

The use of *M. spicata* is importantly characterized in several populations, including Moroccan population, which has used the aerial parts (with tea) of this plant since time against several diseases including diabetes, digestive and respiratory disorders, throat ailments, and skin disease [1, 2].

Certainly, *M. spicata* contains molecules biologically active having biological effects, and effective spectroscopic analysis of extracts and essential oils of *M. spicata* using GC-MS, HPLC, HPLC-MS, and RMN revealed the presence of several phytochemical bioactive compounds belonging to different classes of secondary metabolites in particularly the classes of flavonoids, phenolic acids, and terpenes [3, 4]. Indeed, the distribution of these chemical compounds between different plant parts and collection regions is variable, which explains different traditional uses (with efficacy) of this species according to each region. In addition, the extraction of these chemical compounds depends on used methods and therefore can justify the difference in traditional applications according to used methods of pharmaceutical formulations preparation.

In vitro and in vivo experimental explorations showed that *M. spicata* extracts and essential oils exhibit remarkable biological activities, including antimicrobial, antiparasitic, antidiabetic, anti-inflammatory, and anticancer effects. Indeed, different organic extracts (rich in bioactive compounds) revealed important antifungal activity by their potency to inhibit the growth of some strains involved in human infections such as *Aspergillus niger, Candida albicans, Cryptococcus neoformans*, and *Microsporum audouinii* [5]. Moreover, *M. spicata* showed antibacterial properties against various bacterial strains, either clinical or reference [6, 7]. It was also revealed that *M. spicata* extracts target some human complex diseases, including chronic inflammatory diseases, diabetes, and cancers. Plant extracts inhibit or activate targets and/or pathways involved in these pathologies, including membrane receptors, signaling pathways, and molecular targets [8, 9].

To the best of our knowledge, despite numerous investigations that have been carried out until now showing remarkable results, there are now literature reviews exploring *M. spicata* as a source of potential lead compounds. Therefore, this review aims to explore, discuss, and highlight all data concerning *M. spicata* and give suggestions about its exploitation as a source for developing bioactive compounds in the pharmaceutical and cosmetic fields.

## 2. Research Methodology

## 3. Results and Discussion

### 3.1. Taxonomy, Botanical Description, Geographic Distribution, and Ecological Factors

*Mentha spicata* (ID: 29719) is also known as spearmint. There are a couple of heterotypic synonyms for this species including *Mentha cordifolia, Mentha crispa* var. *crispata f. reticulata, Mentha viridis* (L.) L., *Mentha* *×* *cordifolia*, and *Mentha* *×* *villosa* var. *cordifolia.* It is an aromatic plant that belongs to the genus *Mentha*, family Lamiaceae, subfamily Nepetoideae, placed in Magnoliopsida class, and belongs to order Lamiales. The genus *Mentha*, one of the most important members of the Lamiaceae family, is represented by 19 species and 13 natural hybrids, and Lamiaceae family consists of over 7000 species and around 260 genera of trees and shrubs [10]. The spearmint, *M. spicata*, is a hybrid of *M. longifolia* and *M. rotundifolia.* This species is widely grown in Europe, North America, and Asia, but nowadays cultivated throughout all regions of the world [11].

*M*. *spicata* L. (spearmint) is a creeping rhizomatous, glabrous, and perennial herb with a strong aromatic odor, growing up to 30–100 cm tall with variably hairless to hairy stems and foliage, and a wide spreading fleshy underground rhizome [12]. The leaves are ovate to lancolate, 5–9 cm long and 1.5–3 cm broad, with a serrated margin. Spearmint produces flowers in slender spikes, each flower pink or white, and 2.5–3 mm long and broad. The stem is square-shaped, a trademark of the mint family of herbs [13]. *M*. *spicata* L is well adapted to climatic conditions in tropical and subtropical areas. It can be cultivated in wide range of soils and found in back gardens of homesteads [14].

### 3.2. Medicinal Uses

*Mentha viridis* is widely used in a variety of applications [15]. Since ancient times, Western and Eastern cultures have practiced *Mentha viridis* as a medicinal and aromatic plant against several diseases (Table 1) [15]. Ethnobotanical investigations into *Mentha viridis* have suggested its potential medical applications in different disorders. It has beneficial effects on diabetes, digestive, skin, and respiratory disorders [1, 2, 16–23].

In Morocco, *Mentha viridis* is a medicinal plant most used in the treatment of throat ailments. The use of this plant to treat throat ailments has been demonstrated by Orch et al. [20], who reported the use of aerial parts' infusion of *Mentha viridis* in Moroccan oriental folklore. The leaves of *M. viridis* are also administered as a decoction to treat diabetes in the Al Haouz-Rhamna region (Morocco) [1]. Idm' hand et al. [17] showed that the leaves and stems of *M. viridis* are also used as a decoction and infusion to treat diabetes; on the other hand, El-hilaly et al. [16] showed that these parts were used to treat headache and tiredness. The leaves and flowers of *M. viridis* have also been widely used to treat asthma, bronchitis, chest pain, lung disorders, kidney problems, and diuretics by decoction or infusion [18]. In addition, the leaves of *M. viridis* have been used against gastric disorders by decoction, and the stems are used against ailments of intestines [2, 23]. *M. viridis* whole plant infusions are also used to treat aphrodisiac, cold, flatulence, headache, tonic, and toothache [19, 22]. In another study in Morocco, the powder from the leaves of *M. viridis* is used to treat skin diseases [21].

### 3.3. Phytochemical Compounds

**Figure 1:**
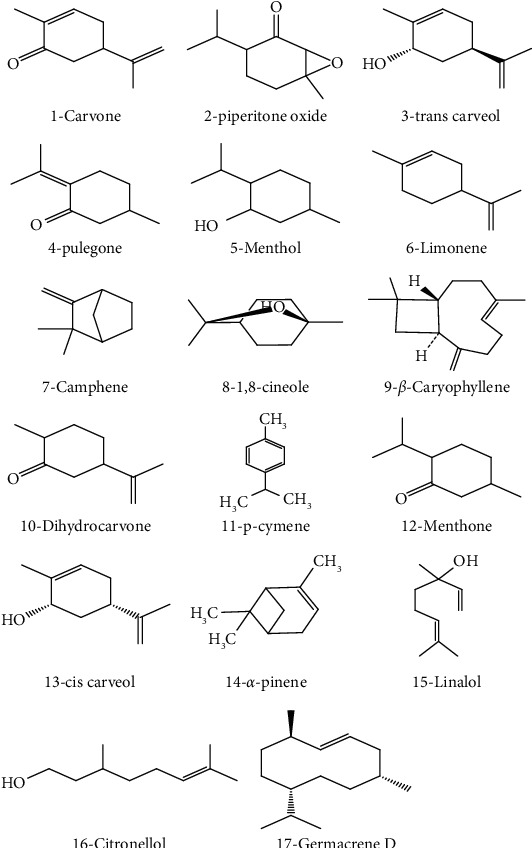
Chemical structures of terpenoids identified.

Extracts and essential oils extracted from *M. spicata* (*viridis*) are considered as valuable source phytochemicals, including natural phenolics and EO_S_. These volatile compounds are complex mixtures of substances that have been found to create different chemotypes distinguished based on the dominant compound in the essential oil, which depends on the plant species, and within the same variety, the essential oil composition can vary according to the geographical region [24]. In terms of phytochemical content, terpenes and terpenoids are the major components of EOs obtained from aerial parts of *M. spicata*. Thus, more detailed discussion regarding chemical aspects of EOs of these species is described (Table 2). Previous studies reported the existence of different chemotypes in the chemical composition of *M. spicata*, naturally grown as cultivated, around the world, and the essential oil mainly composed of carvone, carvacrol, trans-carveol, piperitone oxide, limonene, 1,8-cinéole, camphene, p-cymene, dihydrocarvone, pulegone, *β*-caryophyllene, germacrene D, menthone, *α*-pinene, and linalool [3, 5, 26, 27]; whereas, carvone is mentioned as the absolute predominant constituent of *M. spicata* oil as well as monoterpenes including linalool, piperitone, piperitone oxide, menthone, isomenthone, and pulegone (Figure 1 and Table 2). The composition of *M. spicata* EO_S_ from Morocco is relatively stable and has strong homogeneity [7, 40, 53, 56]. No significant difference between samples was observed; whatever the locality (region), the main essential oil compounds are carvone and trans-carveol, showing variation in a narrow range of 29–47.3% and 14–20%, respectively [34, 46, 47, 51, 52]. Various chemotypes of *M. spicata* were also identified for plants cultivated in Italy and Turkey. In plants from Italy, carvone (39.13–59.26%) was detected as the main compound [29], while for the species from Turkey, piperitenone oxide (25.84%), pulegone (24.72%), cis-piperitenone oxide (12.55%), and limonene 1.59% were the principal constituents of the EO_S_ [31]. It is worth noting that chemotype carvone represented the most variation, 79.70% in spearmint *M. spicata* EO_S_ [24]. Other examples of *M. spicata* producing EOs with high piperitone oxide content (above 70%) are samples from India [36]. As established in the literature, such compound is one of the most abundant components of *M. spicata* EO, which offers spearmint its unique smooth characteristic scent [57], and it also varies according to the spearmint oil grown in different countries. Similarly, EOs from *Cyprus* is reported to possess a higher carvone content (69.23–74.27%) [55].

However, four chemotypes of *M. spicata* were found in Brazil, characterized by the dominant occurrence of carvone which vary from 39.42% to 72.28% and piperitone presented high level 81.18% [7, 56], Although carvone was constantly present as a chief component among spearmint species, there was one landrace with linalool content up to 58.51%. Since all the studies were carried out in the same environmental conditions, this variation may be triggered by their different genetic backgrounds, having evolved due to complex geographic-environmental differences across Brazil. Interestingly, in most *M. spicata* EOs, carvone is the major constituent, notably found in quantities above 50% in EOs extracted from plants cultivated in Hungary, Iran, Bangladesh, Serbia, Czech Republic, and Pakistan [3, 5, 40, 46, 47, 52, 53].

Furthermore, the occurrence of huge chemical variations among *Mentha* accessions collected from diverse countries seems to be due to the divergent climatological and geographical conditions; existing variations in oil content and composition may be attributed to factors related to ecotype and the environment including temperature, relative humidity, irradiance, and photoperiod [34]. Additionally, the reported yields of carvone for *M. spicata* range from 39.21% to 75.53%, being the highest value found for plants cultivated in Tunisia [51].

As given in Table 2, plants cultivated in several states in Iran usually produce EOs with high (>50%) 1,8-cineole content [39]. Similarly, *M. spicata* populations in China also show certain stability in essential oils, with carvone chemotype affording high yield 46.7–65.4% above, while dihydrocarveol acetate (0.2–7%) observed in Chinese spearmint is the only oxygenated sesquiterpenes [46]. Also, a large chemical variability is observed among *M. spicata* essential oil extracted by different methods. Such variation can be attributed to several factors, including genetic, environmental, and their interaction effects, such as plant part, harvest time, extraction method, ecotype, and geographic origin (climate, edaphic, elevation, and topography) [4].

**Figure 2:**
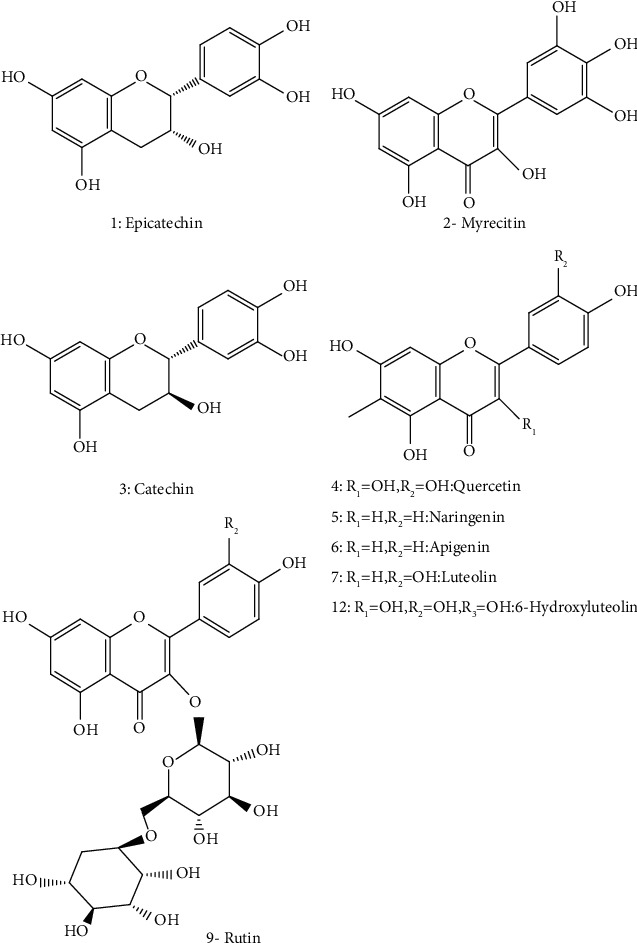
Structures of some flavonoids identified in *M. spicata*.

*M. spicata* has a broad spectrum of bioactive compounds; preliminary screening of *M. spicata* revealed the presence of polyphenols, flavonoids, tannins, sterols, triterpenes, and glycosides [58]. Besides, the chemical composition of *M. spicata* methanolic extracts harvested from different regions of India confirmed the presence of alcohols, phenols, alkanes, alkenes, carbonyl, carboxylic acids, and aromatic compounds [35]. Besides, Bimakr et al. identified the flavonoid content from *M. spicata* leaves by using conventional Soxhlet extraction (CSE) and supercritical carbon dioxide (SC-CO2) extraction [42]. The highest content was obtained with methanol solvent, which extracted seven flavonoids. The highest recovery was recorded for the free aglycone apigenin 27–39.2%, followed by naringenin 5.4–24.9%, epicatechin 15.6–16.3%, catechin 14–14.4%, rutin 14.8–16.1%, myricetin 4.1–11.7%, and luteolin 9.3–65.7% (Figure 2 and Table 2); the same study also identified apigenin as the major isolated flavonoid (6.14 ± 0.76%) from ethanolic and hydroethanolic fractions. Interestingly, supercritical carbon dioxide extract was found to have more main flavonoid compounds and high recovery comparing to the 70% ethanol Soxhlet extraction [42].

**Figure 3:**
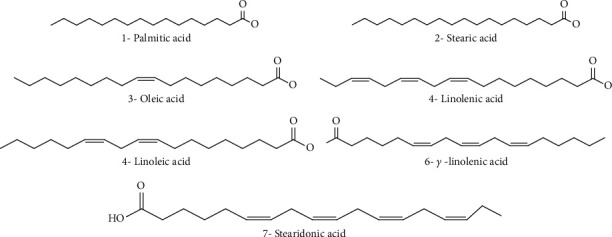
Chemical structures of fatty acids identified in *M. spicata*.

**Figure 4:**
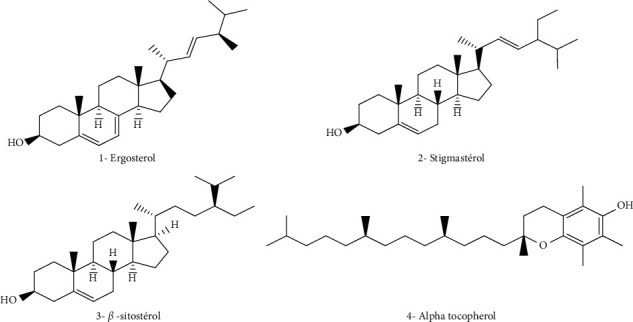
Chemical structures of some sterols identified in *M. spicata* essential oils.

**Figure 5:**
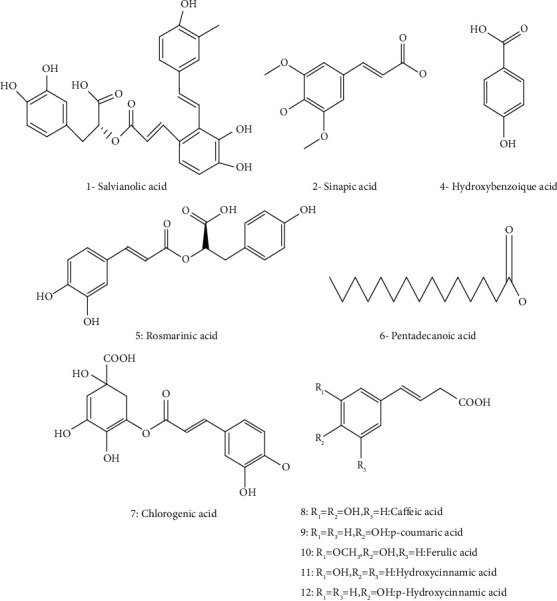
Chemical structures of phenolic acids identified in *M. spicata*.

The ethanolic extracts of *M. spicata* contain a large amount of phenolic compounds (polyphenols, flavonoids, and caffeic acid derivatives); ferulic acid was determined in the highest concentration (27.32%), followed by p-coumaric acid (15.24%) and sinapic acid (6.60%). Caftaric acid, caffeic acid, and chlorogenic acid were also identified in low quantities (Figure 2). In addition, luteolin was identified and quantified (4.68%) in *M. spicata* extract (Figure 2, Table 2) [50]. For fatty acids composition, the EOs produced by *M. spicata* are the most widely investigated among all *Mentha* species. Alpha-linolenic acid (48.17%) has been found to be the major polyunsaturated fatty acid of *M. spicata.* Linoleic acid (31.14%) is the second major polyunsaturated fatty acid in the present study. In comparison, oleic acid (8.19%) and palmitic acid (5.11%) are determined as the major monounsaturated fatty acids, stearidonic acid (3.02%), *γ*-linolenic acid (2.07%), and stearic acid (1.92%) (Figure 3, Table 2). Various phytosterols including ergosterol (51.42%), stigmasterol (7.6%), and beta-sitosterol (2.86%) (Figure 4) have been found in *M. spicata* [33]. Moreover, *M. spicata* contains r-tocopherol (6.11%) and vitamin D3 (31.74%) as lipide-soluble vitamins (Table 2). On the other hand, naringenin (55.44%), naringin (25%), and quercetin (19.38%) have been identified as the major flavonoids in the seeds of *M. spicata*; while, myricetin and catechin constituents are not detected [33] (Figure 5 and Table 2). Polar extracts of spearmint leaves are characterized mainly by a high content of phenolic compounds; the sum of rosmarinic acid and its derivatives was about 88% of the total amount of detected phenolics, followed by salvianolic acids (5.6%) and caffeoylquinic acids (1.2%). Hydroxycinnamic acids, including caftaric acid, represented about 1.1% of total phenolics. All other detected phenolic groups, such as flavonols, flavanones, flavones, hydroxybenzoic acids, and hydroxyphenyl propanoic acids, represented approximately 1% [30].

### 3.4. Mineral and Heavy Metal Contents

Mint tea may be an important source of macro and micrometallic elements, which are essential for human health. However, literature reflects enormous variability in determined concentrations. Indeed, Subramanian et al. [59] revealed that total metal concentrations of Fe, Na, Mg, Mn, Pb, Cd, Cu, and Zn in *Mentha spicata* were 395.74 ± 4.09 mg/kg, 808.09 ± 1.64 mg/kg, 532.72 ± 0.93 mg/kg, 85.72 ± 1.13 mg/kg, 9.89 ± 0.36 mg/kg, 0.74 ± 0.07 mg/kg, 29.83 ± 3.16 mg/kg, and 49.76 ± 4.12 mg/kg, respectively. In another study, Choudhury et al. [60] analyzed ten *Mentha spicata* leaves samples collected from four different locations in Northwest India for minor and trace elements including heavy toxic metals using thermal neutron activation analysis (TNAA) and atomic absorption spectrophotometry (AAS). The authors revealed that the most elements were found in widely varying amounts depending on the location: Na (0.21–0.86 mg/g), K(12.4–53.3 mg/g), and Ca (5.82–16.8 mg/g); whereas, mean contents of other nutrient elements in mint were as follows: Fe (108 ± 22 *μ*g/g), Mg (4.83 ± 0.92 mg/g), Mn (53.5 ± 9.6 *μ*g/g), P (3.88 ± 0.94 mg/g), Cu (16.9 ± 1.8 *μ*g/g), Zn (21.0 ± 4.7 *μ*g/g), and Se (0.18 ± 0.03 *μ*g/g). The toxic heavy metals such as Hg (97–983 ng/g), Sb (1.8–315 ng/g), Ni (0.37–3.22 ng/g), Cd (15–772 ng/g), and As (98–320 ng/g) are all found at ng/g level only but vary in a wide range. Moreover, aerial parts *M. spicata* from Iran contains 129.76 *μ*g/g of Fe, 8.52 *μ*g/g of Zn, and 6.8 *μ*g/g of Mn [61].

### 3.5. Pharmacological Properties of *M. spicata*

**Figure 6:**
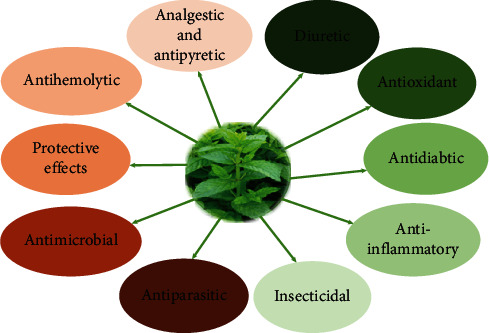
Biological and pharmacological properties of *Mentha spicata*.

*M. spicata* essential oils and extracts exhibit different biological and pharmacological properties (Figure 6). These properties will be discussed in the following sections.

#### 3.5.1. Antifungal Activity

Several studies investigated the antifungal activity of *Mentha spicata* extracts using different parts of the plant and different methods such as the disc diffusion method, microdilution method, agar well diffusion method, spots method, and microdilution broth susceptibility assay [5, 11, 62, 63].

Table 3 provides all studies that examined the antifungal potential of *M. spicata* extracts, showing the type of extract, plant part used, used method, tested strains, and key results. Using the disc diffusion method, Alaklabi et al. [62] assessed the antifungal activity of hexane, chloroform, ethyl acetate, methanol, ethanol, toluene, n-butanol, n-propanol, isopropanol, and water extracts from the root of *M. spicata* against *Aspergillus niger, Candida albicans, Cryptococcus neoformans*, and *Microsporum audouinii*. Water extract showed the highest activity against *M. audouinii* (MIC: 16 *μ*g/mL). It revealed a remarkable antifungal response against other fungal species, *A. niger* (MIC = 32 *μ*g/mL), *C. albicans* (MIC = 64 *μ*g/mL), and *C. neoformans* (MIC = 32 *μ*g/mL). Hexane, chloroform, and ethyl acetate extracts exhibited high antifungal activity against *M. audouinii* with a MIC equal to 32 *μ*g/mL, 64 *μ*g/mL, and 32 *μ*g/mL, respectively. In contrast, the same extracts did not show a significant effect against the other fungal strains tested. Moreover, *C. albicans* was significantly inhibited by toluene and n-butanol extracts (MIC = 64 *μ*g/mL), whereas the fungal activity of *A. niger* was highly reduced by using methanol and ethanol extracts (MIC = 64 *μ*g/mL). Using the same method to screen the antifungal activity of *M. spicata* root extracts, isopropanol extract was found to be less active for the four fungal strains evaluated [62].

To investigate the antifungal properties of essential oil isolated from the aerial parts of *M. spicata* cultivated in the Algerian Saharan Atlas, the results published by Bardaweel et al. [48] showed a lower activity of essential oil of *M. spicata* against *Candida glabrata* (MIC = 256 *μ*g/mL) by employing the microdilution method. Nevertheless, in the Turkish study conducted by Bayan et al. [64], the volatile oil from *M. spicata* extracted of aerial parts exhibited a strong fungitoxicity effect with 100% of inhibition of mycelium growth in *F. oxysporum* f.sp. *radicis-lycopersici* (FORL), *Verticillium dahliae* Kleb (*V. dahliae*), *Alternaria solani* (*A. solani*), and *Rhizoctonia solani* J.G. Kühn. (*R. solani*) at a dose of 12 *μ*L petri^−1^ by using the agar well diffusion method.

In another study from Pakistan, Hussain et al. [5] evaluated the antifungal activity of essential oil of spearmint (*Mentha spicata* L.) isolated from dried aerial parts against five fungal strains. The results showed that *Aspergillus niger* was the most responsive fungal species presenting the largest zone of inhibition (26.9 mm) with the MIC value of 0.07 mg/mL, followed by *Mucor mucedo* (Ф = 26.2 ± 0.8 mm and MIC = 0.08 ± 0.00 *μ*g/mL), *Rhizopus solani* (Ф = 26.3 ± 0.8 mm and MIC = 0.09 ± 0.00 *μ*g/mL), and *Fusarium solani* (Ф = 25.2 ± 1.0 mm and MIC = 0.09 ± 0.00 *μ*g/mL). However, *B. theobromae* was observed to be the most resistant fungus with the smallest inhibition zone (23.0 mm) and a MIC value equal to 0.11 mg/mL by using microdilution broth susceptibility assay.

Additionally, Kedia et al. [66] tested the antifungal potency of essential oil of spearmint against 19 food-deteriorating molds using the poisoned food assay. The fundings showed that the oil of *M. spicata* has a notable potential to inhibit the fungal growth of all fungi species, causing 100% of mycelial inhibition at 1.0 *μ*L ml^−1^ excluding *Aspergillus luchuensis* and *Aspergillus terreus*, where the percentage of mycelial inhibition was 91.72 ± 0.36% and 75.67 ± 0.74%, respectively. The results of testing the nature toxicity of the oil from *M. spicata* revealed that spearmint essential oil possessed a fungicidal effect in *Cladosporium cladosporioides*, *Mycelia sterilia*, *Alternaria alternata*, and *Curvularia lunata* at 1.0 *μ*L mL^−1^. In their study, Liu et al. [11] investigated the biological properties of the essential oil isolated from aerial parts of *M. spicata* from China. Using the disc diffusion method, the results of this study showed quite strong antifungal potency against *A. niger* with an MIC value of 6.25 *μ*g/mL and an MBC value of 12.50 *μ*g/mL. Compared to a study carried out by Şarer et al. [67] from eastern Turkey, the oil of *M. spicata* subsp. *spicata* exhibited high antifungal activity against *Candida albicans* and *Candida tropicalis* with an MIC value less than 3.19 *μ*g/mL.

Regarding testing the potential antimicrobial effects of *M. spicata*, [45] investigated the essential oil extracted from air-dried leaves of Algerian spearmint against *Candida albicans* (ATCC 1024) strain and two *Aspergillus* species (*flavus* NRRL 391 and *niger* 2CA 936). Using the spots method, their finding indicates that *Candida albicans* (ATCC 1024) was the most sensitive species with a diameter of growth inhibition zones equal to 44.3 ± 1.1 mm, followed by *A. flavus* NRRL 391 (Ф = 43.7 ± 0.6 mm), and *A. niger* 2CA 936 (Ф = 36.0 ± 1.0 mm). The disc diffusion method also showed high activity against *Aspergillus* species, *A. flavus* NRRL 391 (Ф = 36.0 ± 2.0 mm) and *A. niger* 2CA 936 (Ф = 32.0 ± 1.0 mm) than *C. albicans* (ATCC 1024) (Ф = 23.3 ± 0.6 mm).

On the other hand, Ojewumi et al. [63] demonstrated the antimicrobial role of the leaf oil extract of *M. spicata* from Nigeria by using two types of petroleum ether and hexane extract. They found that the hexane extract showed higher activity against *Aspergillus niger* (Ф = 26 mm) followed by *Saccharomyces cerevisiae* (Ф = 25). In addition, they observed that petroleum ether extract showed potent activity against *Aspergillus niger* (Ф = 27 mm) followed by *S. cerevisiae* (Ф = 27). Therefore, it was noted that the effectiveness of the two extracts was significantly comparable as the inhibitory zone values are very similar. Furthermore, the ethanolic extract exhibited 100% of inhibition against *Fusarium oxysporum* f.sp. *lentis* in the investigation performed by Singh et al. [68] that aimed to study the antifungal activity of *M. spicata*. The results found were supported by the study conducted in Sudan by Sulieman et al. [69]; they indicated that spearmint oil leaves have demonstrated potent activity against *Aspergillus niger* (ATCC 9763) with an inhibition zone equal to 19 mm at a high concentration (20%) and (15 mm) at low concentration (5%). In addition, the oil of *M. spicata* exhibited considerable inhibition capacity against *C. albicans* with an inhibition zone diameter of 18 mm at higher concentration (20%) and 14 mm at lower concentration (5%). Similarly, the concentration of 100 mg/mL was able to inhibit *C. albicans* with a diameter of growth inhibition zone reached 16 mm using the agar diffusion method [71].

Zaidi et al. [70] evaluated the antifungal efficiency of oil leaves from *M. spicata* against four fungal species including *A. niger* and *Aspergillus* spp., *C. albicans,* and *Rhizopus nigricans*, using the agar well diffusion method. The results showed that *Mentha spicata* oil exhibited an excellent potential against fungal strains tested but with differing sensitivity. *A. niger* showed a strong inhibition zone of 15.7 ± 0.09 mm compared to *C. albicans*, which possessed an inhibition zone of 11.8 ± 0.10 mm. However, *M. spicata* oil was not able to inhibit the growth of *R. nigricans* strain. The oil also exhibited an antifungal effect against *Aspergillus* spp. (13 ± 0.13 mm). In another study, using the agar well diffusion method, essential oil isolated from spearmint was observed to act as a stronger bioactive source against fungal species with a different zone of inhibition. Indeed, inhibition zone diameters for *Aspergillus ochraceus* (NRRL 3174) (Ф = 43 mm) and *Mucor ramamnianus* (ATCC 9314) (Ф = 40 mm) were higher than inhibition zone diameters for *S. cerevisiae* (ATCC 4226 A) (Ф = 25 mm) and *C. albicans* IPA 200 (Ф = 21 mm) [72].

#### 3.5.2. Antibacterial Activity

For over 60 years, antimicrobial agents have been used to treat infections in humans, animals, and plants. Currently, they are among the most widely used therapeutic agents in human and veterinary medicine [73]. At the start of antibiotic therapy, as resistant strains were low and highly effective antimicrobial agents of different classes were detected, antimicrobial resistance was not considered a major problem. This has forced sensitive bacteria living in close contact with antimicrobial producers to develop mechanisms to bypass the inhibitory effects of antimicrobial agents (Table 4). In the context of this study, several in vitro studies have determined the antibacterial activity of *M. spicata* essential oils and solvent extracts against various bacterial strains, either clinical or reference, using the agar diffusion methods (disks or well) and the agar and broth dilution methods [5, 74, 89]. In most of these studies, qualitative inhibition was determined by the dilution method, which is used to assess minimum inhibitory concentration (MIC) and minimum bactericidal concentration (MBC) values [5, 74, 89]. Indeed, the increased selective pressure imposed by the widespread use of antimicrobial agents has clearly accelerated the development and spread of bacterial resistance to antimicrobial agents [5, 74, 89]. These observations underscore the enormous flexibility of bacteria to resist less favorable environmental conditions by constantly developing new survival strategies.

#### 3.5.3. Antiparasitic Activity

Table 5 provides investigations interested in the antiparasitic effect of spearmint [90, 91]. Zandi-Sohani and Ramezani [90] investigated the antiparasitic effect of essential oil isolated from spearmint leaves collected from southwestern Iran against *Tetranychus turkestani*. They discovered that the essential oil of spearmint exhibited acaricidal potential and can be employed to protect against *Tetranychus turkestani*, which showed to cause 100% adult mortality at a concentration of 20 *μ*L/L. The lethal concentration values (LC_50_ and LC_95_) for essential oil spearmint were estimated to be 15.3 *μ*L.L^−1^ and 23.4 *μ*L.L^−1^, respectively. However, the study conducted by Koumad and Berkani [91] demonstrated that spearmint leaves revealed the lowest acaricidal activity against *Varroa destructor* by smoke. Results showed that spearmint killed 26.20% of *Varroa destructor* and reduced the infestation rate by 2.35%. The mortality rate was estimated at 30.65%, and infestation rate was 13.18%.

#### 3.5.4. Insecticidal Activity

Several investigations reported that extracts and essential oils from *M. spicata* have insecticidal activities against some pathogenic microorganisms [3, 92, 93] (Table 6).

Brahmi et al. [65] studied the impact of essential oil from *M. spicata* leaves against *Rhyzopertha dominica.* This study revealed that the essential oil from *M. spicata* leaf was effectively toxic against *Rhyzopertha dominica* adults. At a high concentration of 2 *μ*L/mL, *M. spicata* oil showed high repellent activity against *Rhyzopertha dominica* (56.2% at 30 minutes), and the mortality rate was 43% after 96 hours of treatment. Furthermore, the toxicity contact assay showed that spearmint oil showed a low insecticidal effect with DL_50_ equal to 6.1 *μ*L/mL. In another study, Kedia et al. [66] discovered the possibility of using essential oil extracted from aerial parts of *M. spicata* as a pesticide against the insect pest *Callosobruchus chinensis*. According to their findings, treatment with essential oil from *M. spicata* caused 100% mortality to *C. chinensis* after 12 h at a concentration of 0.1 *μ*L/mL air using the fumigation toxicity test, and 100% repellency was observed at 0.025 *μ*L/mL oil concentration in air during repellent activity assay. Using the probit model, the LC_50_ and LC_90_ values obtained were 0.003 and 0.005 *μ*L/mL air concentrations, respectively. Furthermore, the essential oil from *M. spicata* at 0.1 *μ*L/mL concentration has been reported as the effective fumigant with an oviposition deterrence value estimated at 98%.

In an effort to identify biopesticides for granary weevil to avoid losses of crops caused by insects, Lamiri et al. [94] screened a variety of essential oils for their pesticide effects against *Sitophilus granarius*. They discovered that essential oil of spearmint caused 80% and 43% mortality after 24 h and 48 h of exposure, respectively. These findings indicate that the rate of adult mortality rises as the concentration of oil used in the test increases. The study by Papachristos and Stamopoulos [95] assessed the repellent effects of essential oil extracted from whole flowering plants of spearmint against *Acanthoscelides obtectus*. The results showed that this oil exhibited a highly toxic effect in both males and females with LC_50_ values of 1.2 mL/L air for males and 4.4 mL/L air for females, where males are more affected than females. Also, the oil of spearmint exhibited the most repellent property against *Acanthoscelides obtectus* and appears to be more promising for potential use against this pest.

Abdel-Shafy and Soliman [96] in their research hypothesized that essential oil of spearmint (*M. viridis*) possesses the toxicity effect against embryonated eggs, larvae, and fed females of the cattle tick *Boophilus annulatus* (Acari: Ixodida: Amblyommidae) in Egypt. It was found that oil spearmint (*M. viridis*) was less toxic on embryonated eggs (LC_50_ = 1.20%) as well as on unfed larvae (LC_50_ = 0.90%) and fed females (LC_50_ = 10.57%) than other oils tested, including peppermint (*Mentha piperita*), marjoram (*Majorana hortensis*), lavender (*Lavandula officinalis*), and sweet basil (*Ocimum basilicum*). Compared to the study performed by Derbalah and Ahmed [92], spearmint oil leaf was highly effective against *Callosobruchus maculatus* with an LC_50_ value of 235 ppm. The results showed that oil spearmint could be used as a botanical product to control *C. maculatus* insect in cowpea seeds.

Pavela et al. [3] showed the effects of a variety of essential oils from the genus *Mentha* L., including *M. spicata,* against the larvae and adults of *Culex quinquefasciatus* Say (Diptera: Culicidae). Their finding indicates that the oil of *M. spicata* revealed lower larvicidal efficacy against *C. quinquefasciatus* compared to other oils tested. The lethal response of the oil towards the larvae for LC_50_ was estimated as 92 mg/L and for LC_90_ was estimated as 160 mg/L. Similarly, the study carried out by Govindarajan et al. [38] focused on the possible larvicidal properties of essential oil from *M. spicata* against three larvae species: *A. stephensi, C. quinquefasciatus*, and *A. aegypti*. After the exposure of treatment (24 h), the essential oil from *M. spicata* leaves showed a significant larvicidal effect against *A. stephensi, C. quinquefasciatus*, and *A. aegypti*, with LC_50_ and LC_90_ values of 49.71 versus 100.99 ppm, 62.62 versus 118.70 ppm, and 56.08 versus 110.28 ppm, respectively. Also, the essential oil of *M. spicata* caused 99.6 ± 1.6% mortality for *A. stephensi* and 98.1 ± 1.2% for both *C. quinquefasciatus* and *A. aegypti* at a concentration of 125 ppm.

To test the application for alone or combined, three essential oils were isolated from three medicinal plant species belonging to the *Mentha* genus to manage the rice weevil *Sitophilus oryzae* (Curculionidae). The study conducted by Haouel-Hamdi et al. [93] showed that binary combined Tunisian spearmint oils from *M. rotundifolia, M. viridis*, and *M. longifolia* leaves have exerted an important anti-insecticide activity against *Sitophilus oryzae.* However, *Mentha* essential oils alone revealed the lowest repellent activity to *S. oryzae* adults. After 24 days of exposure, LC_50_ and LC_95_ values of fumigant toxicity of *M. viridis* essential oils alone was 100.16 *μ*L/L air and 192.197 *μ*L/L air, respectively, against *S. oryzae* adults. In addition, the LT_50_ value was 45.52 h for *M. viridis*, and the percentage of mortality was 22% at a concentration of 71.43 *μ*L/L air.

#### 3.5.5. Anti-Inflammatory Activity

Table 7 provides studies focused on the anti-inflammatory propriety of the *M. spicata* in different in vivo experiments [97, 99]. Using the carrageen-induced paw edema method, Yousuf et al. [97] showed that methanol extract from the whole plant of *M. spicata* exhibited a strong anti-inflammatory activity which presenting at both doses 250 and 500 mg/kg of methanol extract a significant dose-dependent reduction of paw edema. Furthermore, the anti-inflammatory action of the extract remained significant until the 6^th^ hour of the test. In another study, Arumugam et al. [98] evaluated in vivo anti-inflammatory effect of different solvent fractions of the ethanolic extract of the dried leaves of *M. spicata* on rats with acute and chronic inflammation by using two experimental approaches, carrageenan and cotton pellet-induced inflammation models. The finding showed that ethyl acetate extract and aqueous fraction were potent in cotton pellet (chronic) induced inflammation where the rate of inflammation was reduced by 65% and 54%, respectively. However, inflammation was reduced with less effectiveness in hexane extract (0–20%) and aqueous fraction (7–11%); only the ethyl acetate fraction was found to be effective in carrageenan (acute) induced inflammation, while chloroform fraction has not been able to decrease inflammation.

The study conducted by Jabbar and Kathem [99] evaluated the preventive effect of ethanolic extract of leaves from *M. spicata* on irinotecan-induced mucositis in mice. The results revealed that the ethanolic extract of *M. spicata* markedly reduced jejunal tissue IL-1*β* (3.47 ± 1.23 vs. 6.5 ± 0.36 ng/mL), and fecal *β-*glucuronidase activity (79.78 ± 10.7 vs. 120.6 ± 8.3 U) compared to no-treated mice. In addition, histological investigation of the jejunum section of the animal after administration of irinotecan and ethanolic extract of *M. spicata* showed enhancements in mucositis features.

#### 3.5.6. Antidiabetic Activity

Diabetes mellitus is a metabolic disease that affects the endocrine system, often occurring when the pancreas does not secrete enough insulin or when the body cannot use this hormone effectively, resulting in chronic hyperglycemia with disruptions in protein, lipid, and carbohydrate metabolism.

In order to understand the mechanism of antidiabetic action of *M. spicata* better, several recent studies (in vivo and in vitro) performed in chronological order were discussed in this review [8, 100, 101] (Table 8).

Regarding in vivo studies, Al-Fartosi and collaborators evaluated this activity on male rats rendered diabetic by alloxan intraperitoneal injection (125 mg/kg b.w) and treated with phenolic compounds (200 mg/kg b.w) extracted from the leaves of this plant [100]. During 14 days of daily treatment, a decrease in the level of blood glucose, triglycerides, cholesterol, plasma LDL, and VLDL and a significant increase in plasma HDL levels were recorded. This work confirmed the potential of *M. spicata* in the management of diabetes and its complications. In 2017, two similar studies verified these findings on the same animal model. Indeed, the aqueous ethanolic extract (200 and 400 mg/kg b.w) [101] and the aqueous extract (300 mg/kg b.w) [13] of the leaves of this species presented the same results as the previous study. The following year, 40 streptozotocin-induced diabetic rats were treated for 4 weeks with butanol extract from *M. spicata* roots [8]. At the end of this period, the authors observed antidiabetic properties represented by a decrease in blood glucose level and an increase in bodyweight.

A very recent investigation tested this powder on two carbohydrate hydrolyzing enzymes, namely, *α*-amylase and *α*-glucosidase [86]. In fact, inhibiting these two enzymes prevents the digestion of carbohydrates, which is a promising strategy in the treatment of diabetes. The results of this study showed that the leaf essential oil of this herb at doses of 200 and 250 *μ*L was able to inhibit *α*-amylase (IC_50_ = 101.72 ± 1.86 *μ*g/mL) and *α*-glucosidase (IC_50_ = 86.93 ± 2.43 *μ*g/mL), respectively.

From these studies, it can be inferred that *M. spicata* may be used as an antidiabetic agent; however, further investigations, as well as clinical trials, must be carried out to evaluate this benefit in humans.

#### 3.5.7. Antioxidant Activity

Oxidative stress corresponds to an attack on cells by free radicals, also called reactive oxygen species (ROS), produced continuously from oxygen in the cell, particularly in the mitochondrial respiratory chain. ROS are reactive and very toxic substances. Oxidative stress is caused by an imbalance between the production of prooxidant free radicals and antioxidants. Regarding *M. spicata*, many studies have evaluated its antioxidant activity either by measuring its effectiveness in scavenging free radicals or by directly assaying the products formed using photometric techniques [5, 78, 102] (Table 9). Indeed, Getahun et al. [78] obtained essential oils by hydrodistillation from *M. spicata* leaves to determine their radical scavenging potentials in vitro in DPPH and deoxyribose degradation assays. These oils exhibited potent radical scavenging activities, with IC_50_ values of 5.96 and 0.57 *μ*L/mL in the DPPH and deoxyribose degradation assays, respectively. In the same year, Nickavar et al. [102] found that the ethanolic extract of *M. spicata* aerial parts showed IC_50_ values of 87.89 and 173.80 *μ*g/mL by the DPPH^•^ and ABTS^•+^ assays, respectively. The following year, using the same methods, Mkaddem et al. [72] showed that the essential oil from the leaves of this plant has significant anti-free radical potential.

By respecting the chronology of the studies carried out over time, Ebrahimzadeh et al. [9] examined the antioxidant capacity of *M. spicata* aerial parts in vitro using eight assay systems. They recorded the best activity with the DPPH test (IC_50_ = 105.8 ± 3.98 *μ*g/mL), followed by the assay of nitric oxide-scavenging activity (IC_50_ = 210.6 ± 7.7 *μ*g/mL) and scavenging of H_2_O_2_ (IC_50_ = 631.1 ± 26.0 *μ*g/mL). In addition, good antioxidant activity has been demonstrated by Hussain et al. [5] (IC_50_ = 13.3 ± 0.6 *μ*L/mL) and by Liu et al. [11] (IC_50_ = 72.07 ± 0.34 mg/mL), using DPPH free radical-scavenging ability. Moreover, the antioxidant power of *M. spicata* aerial parts has been tested by Benedec et al. [50] using only the DPPH radical scavenging assay, which showed a value of 18.34 ± 2.2% at the concentration of 0.4 mg/mL. A Tunisian research team also confirmed this when they recorded an important antiradical (IC_50_ = 10 ± 0.24 *μ*g/mL) and superoxide anion (IC_50_ = 1.33 ± 0.10 *μ*g/mL) scavenging ability [104]. Furthermore, according to Teixeira and collaborators, the essential oil of this plant was shown to be a potent antioxidant by exhibiting a dose-dependent antioxidant effect at the concentrations tested (25, 50, 100, 150, 200, 250, 300, and 500 *μ*g/mL), determined by the sequestration of the DPPH radical and by the *β*-carotene-linoleic acid method [44].

Using the same methods as previous studies, other more recent investigations have confirmed the important antioxidant activity of *M. spicata*, regardless of its harvest region or parts used (Table 9).

The antioxidant activity of different parts of *M. spicata* is certainly attributed to its major compounds. Indeed, L-menthone (32.74%) and pulegone (26.67%) were the main volatiles of its essential oil, while apigenin (38.4 mg/100 g dry weight) was the main flavonoid in methanolic extracts [104]. These molecules are renowned for their antioxidant potential [109].

#### 3.5.8. Diuretic Activity

The in vivo study performed by Aziz et al. [110] assessed the diuretic property of the aqueous methanol extract from aerial parts of spearmint in rat models. The treatment administered to experimental rats at dose 100 mg/kg revealed significant diuresis (3.74 ± 0.41 mL). The values obtained are more or less close to the reference standard (furosemide, 4.05 ± 0.34 mL) (*p* < 0.05). Also, the extract of spearmint significantly increased the excretion of potassium and sodium (*p* < 0.05), while a significant change in the pH has not been observed after administration of *M. viridis* extract.

#### 3.5.9. Analgesic and Antipyretic Activities

For testing the analgesic and antipyretic effects of methanol extract from *M. spicata*, Yousuf et al. [97] in their study demonstrated that the methanol extract from the whole plant of *M. spicata* had markedly increased the reaction time of mice in a dose-dependent manner by the hot-plate test (*p* < 0.001) proving its marked analgesic effect. In addition, using the acetic acid-induced writhing method, the methanol extract of *M. spicata* also exhibited a significant analgesic action. The inhibition at the dose of 500 mg/kg was estimated at 60.30%. On the other hand, using Brewer's yeast-induced pyrexia in rats, the methanol extract of *M. spicata* was revealed to exert a strong marked (*p* < 0.01) antipyretic activity at the dose of 500 mg/kg at 3 h than at a dose of 100 mg/kg at 2 h.

#### 3.5.10. Antihemolytic Activity

In order to investigate the biological functions of *M. spicata*, Ebrahimzadeh et al. [9] decided to study the antihemolytic effect of ethanol-water extract from aerial parts of *M. spicata*. The results showed that this extract possesses a weak inhibiting effect with an IC_50_ = 1250.7 ± 46.1 *μ*g/mL by H_2_O_2_-induced membrane damage and hemolysis.

#### 3.5.11. Protective Effects

In their research, Saad et al. [111] were interested in studying the protective activity of *M. spicata* treatment against nicotine-induced oxidative damage in the liver and erythrocytes Wistar rats. The findings showed that aqueous extract from aerial parts of *M. spicata* exhibited a strong protective action. On the hematological parameters, it was found to restore to normal levels the levels of erythrocytes, haematocrit, hemoglobin, and white blood cells. However, on hepatic dysfunction parameters, the aqueous extract of spearmint significantly decreased ALT and ALP activities resulting in a decrease in liver toxicity. Furthermore, the aqueous extract of *M. spicata* to nicotine-treated rats provided a statistically significant (*p* ≤ 0.01) enhancement of antioxidant enzyme capacities, including CAT, SOD, and GPX activities, suggesting an improvement in antioxidant status. According to liver histological analysis, the treatment with the aqueous extract of *M. spicata* showed considerable recovery in the form of hepatic histoarchitecture. Similarly, Saad et al. [111] aimed to screen the in vivo and in vitro antioxidative effect of *M. spicata* extract against nicotine-induced oxidative injury in the kidney and brain of rats. The in vivo results obtained reported that *Mentha* extract significantly increased the bodyweight of rats as well as exhibited a significant increase in testis, brain, and accessory sex organ weights. In addition, treatment with the aqueous extract of *M. spicata* had a significant decrease in the MDA levels, but no significant changes in brain AChE were recorded. Also, *M. spicata* extract supplementation could restore the antioxidant enzymes activities to normal levels and participate to ameliorate cerebral cortex histological pictures and histological damages.

### 3.6. Toxicity Investigations

In pharmacology, the efficacy of a plant or a natural constituent is not sufficient to justify its therapeutic use. Indeed, each bioactive substance is likely to have deleterious effects for human health, at least in high doses and over long periods [112]. In addition to efficacy, the active dose must be free from any toxicity and demonstrate safety. Therefore, in the therapeutic indication of any substance, it is imperative to define its risk-benefit ratio.

Despite the data paucity on its safety profile and given its wide use, the acute and subacute toxicities of *M. spicata* have been tested in four studies to optimize its use [66, 113, 114] (Table 10).

Initially, Yousuf et al. [97] orally administered single doses of 500, 1000, and 2000 mg/kg of whole plant methanolic extract to mice of both sexes. After 24 hours of observation, no mortality or signs of toxicity were noticed. One year later, aerial parts of the same extract at a dose of 5000 mg/kg of extract (the limit test dose according to OECD guidelines 425) showed similar results in female rats [113]. Indeed, during the 14 days of oral gavage, no mortality was recorded, considering the LD_50_ to be greater than 5000 mg/kg. In addition, no changes in the behavior and the bodyweight of the animals were observed. At the end of the experiment and after sacrificing animals, there were no toxicologically significant biochemical and hematological changes compared to the control group. The histological evaluation did not reveal any morphological changes or gross lesions in the lung, kidney, liver, and heart tissues. These results corroborate those obtained by Kedia et al. [66]. They recorded low toxicity (LD_50_ = 8342.33 *μ*L/kg) of the essential oil of *M. spicata* aerial parts following oral administration of different doses (0.05–0.5 mL) to mice (*Mus musculus* L.).

In the same year, Mugisha and colleagues tested the acute and subacute toxicities of the leaves of this plant in Swiss mice and Wistar albino rats, respectively [114]. For acute toxicity, animals received intragastrically over 72 hours, doses of 10000, 12000, 14000, 16000, and 18000 g/kg b.w of the 70% ethanolic extract. Therefore, a death rate of 100% was obtained at the highest dose with some signs of toxicity (convulsions, abdominal muscle contractions, and hyperurination) above 12000 mg/kg b.w. The LD_50_ value was 13606 mg/kg b.w. Regarding subacute toxicity (28 days), ethanol leaf extract (500, 1000, and 1500 mg/kg b.w) caused no mortality or signs of toxicity. However, it significantly increased the levels of mean corpuscular hemoglobin concentration, lymphocytes, blood cells count, and aspartate transferase and significantly reduced haematocrit. At the same time, serum urea and creatinine levels were not affected, confirmed by histopathological data.

From these toxicological investigations, it can be declared that *M. spicata* is an experimentally safe plant, thus justifying its use in treating numerous abnormalities. However, prolonged treatment in high doses can lead to specific problems. For this, other studies on this plant's chronic toxicity are necessary to complete its toxicological profile.

## 4. Conclusion and Perspectives

In this work, we reported the ethnobotanical, phytochemical, and pharmacological aspects of *M. spicata* (*M. viridis*). This medicinal plant is frequently used in traditional practices to treat certain diseases and showed interesting biological properties in various scientific investigations. Phytochemical studies of this species showed its richness in numerous bioactive compounds in particularly terpenoid components, exhibiting important biological effects. Pharmacological biology explorations demonstrated that extracts and essential oils of *M. spicata* showed different pharmacological properties such as antibacterial, antiparasitic activity, insecticidal, anti-inflammatory, antidiabetic, antioxidant, diuretic, analgesic, antipyretic, antihemolytic, and protective activities. However, these effects were evaluated often using in vitro and in vivo approaches, and therefore, further investigations to validate these activities with determining mechanisms of their actions are needed. Toxicological investigation of *M. spicata* extracts was examined by some studies and showed a safety of this plant. However, clinical trials were not conducted, and there is an urgent need to perform such trials to promote the use of the plant especially after proving its excellent safety profile in the toxicological investigation. Indeed, bioactive compounds of *M. spicata* need further investigations concerning the pharmacodynamic and pharmacokinetic aspects to determine their bioavailability and their mechanisms of action of different targets.

## Figures and Tables

**Table 1:** Medicinal use of *M. spicata*.

Used part	Dosage form	Traditional use	References
Leaf	Decoction	Diabetes	[1]
Leaf	Decoction	Against stomach disorders	[2]
Leaf, stem	Infusion	Headache, tiredness	[16]
Leaf, stem	Infusion and decoction	Diabetes	[17]
Leaf, flower	Infusion and decoction	Asthma, bronchitis, chest pain, lungs disorder, kidney problems, diuretic	[18]
Leaf	Infusion	Cold and flu, toothache	[19]
Aerial parts	Infusion	Throat affection	[20]
Leaf	Powder	Skin diseases	[21]
Whole plant	Infusion	Aphrodisiac, cold, flatulence, headache, tonic, toothache	[22]
Leaf, stem	Decoction	Against the ailments of intestines	[23]

**Table 2:** Chemical compounds of *M. spicata*.

Country	Part used	Compounds	Reference
Morocco	Aerial parts	Carvone (33.14%)	[25]
		trans-Carveol (20.06%)	
		*β*-Caryophyllene (4.41%)	
		1,8-Cineole (3.99%)	
		Germacrene D (3.14%)	
		Menthone (2.19%)	
		*α*-Pinene (1.06%)	
	Aerial parts	Carvone (47.30–69.19%)	[26]
		Limonene (4.48–15.43%)	
		trans-4-Caranone (0.82–4.63%)	
		iso-Dihydrocarveol acetate (0.06–2.66%)	
		*ρ*-Mentha-3,8-diene (0.85–1.32%)	
	Aerial parts	Carvone (57.11%)	[27]
		Limonene (27.77%)	
		3-Carene (1.01%)	
		Germacrene D (0.65%)	
	Aerial parts	Carvone (29.00%)	[28]
		trans-Carveol (14.00%)	
		1,8-Cineole (7.30%)	
		Dihydrocarveol (l4.50%)	
		Carvyl acetate-Z (6.70%)	
		Germacrene D (3.90%)	

Italy	Aerial parts	Carvone (39.13 to 59.26%)	[29]
		1,8-Cineole (1.07–9.02%)	
		Dihydrocarveol (2.36–5.94%)	
		Germacrene D (1.79–4.11%)	
		Limonene (5.9–11.40%)	
		trans-Carvyl acetate (0.72–5.90%)	
	Aerial parts	p-Cymene (33.9%)	[30]
		iso-Piperitone (23.7%)	
		Piperitone (6.9%)	
		Menthone (21.8)	
		p-Cymen-8-ol (19.6)	
		*β*-Linalool (15.2%)	

Czech Republic	Aerial parts	Carvone (0.7–59.1%)	[3]
		Menthol (1.1%–14.9%)	
		p-Menthone (1.1%–4.4%)	
		Piperitone oxide (34.1%)	
		Germacrene D (14.6%)	
		*β*-Caryophyllene (2.2–3%)	
		Dihydrocarvone (11.8–12.7%)	
		cis-Jasmone (1.6–1.8%)	

Turkey	Aerial parts	Piperitenone oxide (25.84%)	[31]
		Pulegone (24.72%)	
		cis-Piperitenone oxide (12.55%)	
		Limonene (1.59%)	
	Aerial parts	Carvone (34.70 to 79.70%)	[24]
		1,8-Cineole (3.40–33.80%)	
		*β*-Pinene (0.87–5.29%)	
		Limonene (1.10–22.10%)	
		Menthone (0.20–2.73%)	
		Pulegone (1.70–9.94%)	
	Aerial parts	Carvone (48.6–57.9%)	[32]
		*ρ*-Cymene (9.6–20.5%)	
		1,8-Cineole (14.6–19.3%)	
		Carvacrol (0.1–3.5%)	
		*α*-Pinene(2.3–4.3%)	
	Phenolic acids	Rosmarinic acid derivatives (88%)	[30]
		Salvianolic acids (5.6%)	
		Caffeoylquinic acids (1.2%)	
		Hydroxycinnamic (1.1%)	
	Fatty acids	Palmitic acid (5.11 0.41%)	[33]
		Stearic acid (1.92 ± 0.21%)	
		Oleic acid (8.19%)	
		Linoleic acid (31.14%)	
		*α*-Linolenic acid (48.17%)	
		*γ*-Linolenic acid(2.07%)	
		Stearidonic acid (3.02%)	

India	Aerial parts	Carvone (49.62–76.65%)	[34]
		Limonene (9.57–22.31%)	
		1,8-Cineole (1.32–2.62%)	
		trans-Carveol (0.3–1.52%)	
	Phenolics	Pentadecanoic acid (7.47%)	[35]
		7-Oxabicyclo[4.1.0] heptane (9.56%)	
		3-Penten-2-one,4-(2,2,6-trimethyl-7-oxabicyclo[4.1.0] hept-1-yl)-,(E)-(12.20%) stigmast-4-EN-3-one (18.99%)	
	Aerial parts	trans-Muurola-4 (14%)	[36]
		5-Diene (27.28%)	
		Piperitenone oxide (22.22%)	
		*β*-Caryophyllene (10.48%)	
		Geranyl propanoate (6.55%)	
		Sibirene (3.45%)	
		Borneol (1.98%)	
		Allo-ocimene (1.71%)	
		*β*-Elemene (1.34%)	
		Germacrene D-4-ol (1.02%)	
	Aerial parts	Carvone (57.49–72.47%)	[37]
		Limonene (10.70–24.81%)	
		Myrcene (0.25–4.36%)	
		1,8-Cineole (0.2–2.02%)	
	Aerial parts	Carvone (48.60%)	[38]
		Limonene (11.30%)	
		cis-Carveol (21.30%)	
		Linalool (1.30%)	
		1,8-Cineole (2.55%)	
		cis-Carvyl acetate (2.10%)	
		cis-Dihydrocarvone (1.30%)	
Iran	Aerial parts	Carvone (65.15–74.21%)	[39]
		Limonene (12.22–20.55%)	
		cis-Dihydrocarvone (2.34–11.13%)	
		Caryophyllene (1.13–5.06%)	
	Aerial parts	Carvone (42.74–54.34%)	[4]
		trans-Dihydrocarvone (21.58%)	
		1,8-Cineole (8.41–21.78%)	
		Pulegone (6.83%)	
		Limonene (5.2–6.1%)	
		*β*-Caryophyllene (3.05%)	
		Linalool (5.82%)	
		trans-Dihydrocarvone (3.18%)	
	Aerial parts	Carvone (49.91%–56.92%)	[40]
		Piperitone oxide (10.69%–11.72%)	
		1,8-Cineole (3.78–3.34%)	
		Limonene (7.33–6.61%)	
		Germacrene D (6.26–1.90%)	
	Aerial parts	Carvone (54.34%)	[41]
		1,8-Cineole (8.41–22.71%)	
		Piperitenone oxide (58.87%)	
		3,8-Menthadiene (21.58%)	
		*α*-Pinene (0.95–1.68%)	
		2-Cyclohexen (42.74%)	
		Borneol (5.82%)	
		DL-Limonene (5.2%)	
		Pulegone (6.83%)	

Malaysia	Flavonoids leaves	Catechin (14–14.4%)	[42]
		Epicatechin (15.6–16.3%)	
		Rutin 1 (4.8–16.1%)	
		Myricetin (4.1–11.7%)	
		Luteolin (9.3–65.7%)	
		Apigenin (27–39.2%)	
		Naringenin (5.4–24.9%)	

Algeria	Leaves	Carvone (59.40%)	[7]
		Limonene (6.12%)	
		1,8-Cineole, germacrene D (04.66%)	
		*β*-Caryophyllene (2.969%)	
		*β*-Bourbonene (2.796%)	
		*α*-Terpineol (1.986%)	
		Terpinene-4-ol (1.120%)	

Brazil	Aerial parts	Carvone (39.42–72.28%)	[43]
		Pulegone (5.53–10.48%)	
		Carveol (3.30–4.98%)	
		Cineol (1.49%)	
	Leaves	Linalool (58.51%)	[6]
		Carvone (15.1%)	
		*α*-Terpineol (1.43%)	
		*β*-Caryophyllene (2.02%)	
		Eucalyptol (1.04%)	
		Terpinen-4-ol (5.73%)	
	Leaves	Piperitone (81.18%)	[44]
		Piperitenone (14.75%)	
		*α*-Pinene (0.51%)	
		Limonene (1.47%)	
	Aerial parts	Limonene (2.04–19.91%),	[45]
		Isomenthone (0.46–11.60%)	
		Menthone (0.46–11.60%)	
		1,8-Cineole (eucalyptol) (2.98–8.10%)	
		d-Carvone (31.35–60.07%)	
		*β*-Pinene (2.41–4.27%)	
		Isomenthone (4.46%)	
		Pulegone (6.68–53.65%)	

China	Aerial parts	Carvone (46.7–65.4%)	[46]
		Limonene (0.3–1.8%)	
		Linalool (0.6 6.9%)	
		Menthone (1.5–4.7%)	
		Dihydrocarvone (0.8–15.7%)	
		Dihydrocarveol acetate (0.2–7%)	

China	Aerial parts	Carvone (65.33%)	[11]
		Limonene (18.19%)	
		Dihydrocarvone (2.97%)	
		Camphene (2.34%)	

Hungary	Leaves	Carvone (35.9–60.5%)	[47]
		Citronellol (10.1–13.4%)	
		Limonene (1.6–9.4%)	
		Menthone (3.2–4.4%)	
		*α*-Terpineol (2.1–3%)	
		cis-Dihydrocarvone (1.5–2.2%)	
		(e)-b-Caryophyllene (1.5–2.1%)	

Jordan	Aerial parts	Carvone (49.5%)	[48]
		Limonene (16.1%)	
		1,8-Cineole (8.7%)	
		cis-Dihydrocarvone (3.9%)	
		*β*-Caryophyllene (2.7%)	
		Germacrene D (2.1%)	
		*β*-Pinene (1.1%)	

Abu Dhabi	Leaves	Carvone (14.79–87.11%)	[49]
		Dihydrocarvone (0.09–0.19%)	
		Cineole (0.2–0.6%)	
		Limonene (1.94–9.72%)	
		Menthol (0.06–0.19%)	
		Linalool (0.09–0.23%)	
		*α*-Pinene (0.05–0.3%)	

Romania	Phenolics, 70% ethanol	Ferulic acid (27.32%)	[50]
		Sinapic acid (6.60%)	
		p-Coumaric acid (15.24%)	
		Luteolin (4.68%)	

Tunisia	Aerial parts	Carvone (39.21–75.53%)	[51]
		1,8-Cineole (7.24–12.49%)	
		Limonene (6.07–18.45%)	
		cis-Dihydrocarveol (1.17–6.56%)	
		trans-Carveol (0–5.22%)	
		Pulegone (38.74%)	
		Menthone (28.56%)	
		Menthol (5.64%)	

Bangladesh	Aerial parts	Carvone (73.29%)	[52]
		D-Limonene (7.59%)	
		Dihydrocarvone (3.83%)	
		*α*-Bourbonene (1.67%)	
		trans-Sabinene hydrate (1.57%)	
		trans-Carveol (1.25%)	
		Dihydrocarveol (1.12%)	
		Eucalyptol (1.01%)	

Serbia	Aerial parts	Carvone (49.5%)	[53]
		Menthone (21.9%)	
		Piperitone 0.6%)	
		*β*-Bourbonene (26.8%)	
		*β*-Caryophyllene (0.7%)	
		Germacrene A(0.5%)	

Palestine	Aerial parts	Limonene (6.23–9.79%)	[54]
		Carvone (36.9–76.82%)	
		Sabinene (0.14–5.51%)	
		cis-Dihydrocarvone (0.65–4.59%)	
		*β*-Caryophyllene (0.81–3.87%)	
		Dihydrocarveol (2.27–13.76%)	

Cyprus	Aerial parts	Carvone (ketone: 69.23–74.27%)	[55]
		Limonene (alkene: 10.42–11.39%)	
		1,8-Cineole (alcohol: 5.28–5.99%)	
		*β*-Pinene (alkene: 1.13–1.25%)	
		*β*-Caryophyllene (alkene: 0.80–1.29%)	
		Germacrene D (alkene: 2.09–3.13%)	
		Bicyclogermacrene (0.60–1.01%)	

Pakistan	Aerial parts	Carvone (51.7%)	[5]
		cis-Carveol (24.3%)	
		Limonene (5.3%)	
		1,8-Cineol (4.0%)	
		cis-Dihydrocarvone (2.2%)	
		Carvyl acetate (2.1%)	
		cis-Sabinene hydrate (1.0%)	

**Table 3:** Antifungal activity of *Mentha spicata*.

Used part	Extracts	Used method	Tested strains	Key results	References
Root	Hexane extract	Disc diffusion method	*A. niger*	MIC > 356 *μ*g/mL	[62]
			*C. albicans*	MIC > 356 *μ*g/mL	
			*C. neoformans*	MIC > 356 *μ*g/mL	
			*M. audouinii*	MIC = 32 *μ*g/mL	
Root	Chloroform extract		*A. niger*	MIC > 356 *μ*g/mL	[62]
			*C. albicans*	MIC > 356 *μ*g/mL	
			*C. neoformans*	MIC = 64 *μ*g/mL	
			*M. audouinii*	MIC = 64 *μ*g/mL	
Root	Ethyl acetate extract		*A. niger*	MIC = 128 *μ*g/mL	[62]
			*C. albicans*	MIC = 128 *μ*g/mL	
			*C. neoformans*	MIC = 128 *μ*g/mL	
			*M. audouinii*	MIC = 32 *μ*g/mL	
Root	Methanol extract		*A. niger*	MIC = 64 *μ*g/mL	[62]
			*C. albicans*	MIC = 128 *μ*g/mL	
			*C. neoformans*	MIC = 128 *μ*g/mL	
			*M. audouinii*	MIC > 356 *μ*g/mL	

Root	Ethanol extract	Disc diffusion method	*A. niger*	MIC = 64 *μ*g/mL	[62]
			*C. albicans*	MIC = 128 *μ*g/mL	
			*C. neoformans*	MIC = 128 *μ*g/mL	
			*M. audouinii*	MIC > 356 *μ*g/mL	
Root	Toluene extract		*A. niger*	MIC > 356 *μ*g/mL	[62]
			*C. albicans*	MIC = 64 *μ*g/mL	
			*C. neoformans*	MIC > 356 *μ*g/mL	
			*M. audouinii*	MIC = 128 *μ*g/mL	
Root	N-butanol extract		*A. niger*	MIC = 128 *μ*g/mL	[62]
			*C. albicans*	MIC = 64 *μ*g/mL	
			*C. neoformans*	MIC > 356 *μ*g/mL	
			*M. audouinii*	MIC = 128 *μ*g/mL	
Root	N-propanol extract		*A. niger*	MIC = 64 *μ*g/mL	[62]
			*C. albicans*	MIC = 64 *μ*g/mL	
			*C. neoformans*	MIC = 32 *μ*g/mL	
			*M. audouinii*	MIC = 128 *μ*g/mL	

Root	Isopropanol extract	Disc diffusion method	*A. niger*	MIC = 128 *μ*g/mL	[62]
			*C. albicans*	MIC = 128 *μ*g/mL	
			*C. neoformans*	MIC > 356 *μ*g/mL	
			*M. audouinii*	MIC > 356 *μ*g/mL	
Root	Water extract		*A. niger*	MIC = 32 *μ*g/mL	[62]
			*C. albicans*	MIC = 64 *μ*g/mL	
			*C. neoformans*	MIC = 32 *μ*g/mL	
			*M. audouinii*	MIC = 16 *μ*g/mL	

Aerial parts	Essential oil	Microdilution method	*Candida glabrata*	MIC = 256 *μ*g/mL	[48]
Aerial parts	Volatile oil	Agar well diffusion method	*R. solani*	Inhibition = 100% at dose of 12 *μ*L	[64]
			*A. solani*	Inhibition = 100% at dose of 12 *μ*L	
			*F. oxysporum* f.sp. *radicis-lycopersici*	Inhibition = 100% at dose of 12 *μ*L	
			*V. dahliae*	Inhibition = 100% at dose of 12 *μ*L	

Leaves	Essential oil	Spots method	*A. niger* 2CA 936	Ф = 36.0 ± 1.0 mm	[65]
			*A. flavus* NRRL 391	Ф = 43.7 ± 0.6 mm	
			*C. albicans* (ATCC 1024)	Ф = 44.3 ± 1.1 mm	

Leaves	Essential oil	Disc method	*A. niger* 2CA 936	Ф = 32.0 ± 1.0 mm	[65]
			*A. flavus* NRRL 391	Ф = 36.0 ± 2.0 mm	
			*C. albicans* (ATCC 1024)	Ф = 23.3 ± 0.6 mm	

Aerial parts	Essential oils	Disc diffusion method Microdilution broth susceptibility assay	*Aspergillus niger*	Ф = 26.9 ± 1.2 mm	[5]
				MIC = 0.07 ± 0.00 *μ*g/mL	
			*Mucor mucedo*	Ф = 26.2 ± 0.8 mm	
				MIC = 0.08 ± 0.00 *μ*g/mL	
			*Fusarium solani*	Ф = 25.2 ± 1.0 mm	
				MIC = 0.09 ± 0.00 *μ*g/mL	
			*Botryodiplodia theobromae*	Ф = 23.0 ± 1.1 mm	
				MIC = 0.11 ± 0.01 *μ*g/mL	
			*Rhizopus solani*	Ф = 26.3 ± 0.8 mm	
				MIC = 0.09 ± 0.00 *μ*g/mL	

Aerial parts	Essential oil		*Absidia ramosa*	Mycelial inhibition = 100%	[66]
			*Alternaria alternata*	Mycelial inhibition = 100%	
			*Aspergillus fumigatus*	Mycelial inhibition = 100%	
			*Aspergillus glaucus*	Mycelial inhibition = 100%	
			*Aspergillus luchuensis*	Mycelial inhibition = 91.72 ± 0.36%	
			*Aspergillus niger*	Mycelial inhibition = 100%	
			*Aspergillus terreus*	Mycelial inhibition = 75.67 ± 0.74%	
			*Aspergillus unguis*	Mycelial inhibition = 100%	
			*Cladosporium cladosporioides*	Mycelial inhibition = 100%	
			*Curvularia lunata*	Mycelial inhibition = 100%	
			*Fusarium oxysporum*	Mycelial inhibition = 100%	
			*Mucor* spp.	Mycelial inhibition = 100%	
			*Mycelia sterilia*	Mycelial inhibition = 100%	
			*Penicillium citrinum*	Mycelial inhibition = 100%	
			*Penicillium italicum*	Mycelial inhibition = 100%	
			*Penicillium luteum*	Mycelial inhibition = 100%	
			*Penicillium purpurogenum*	Mycelial inhibition = 100%	
			*Rhizopus stolonifer*	Mycelial inhibition = 100%	
			*Spondylocladium australe*	Mycelial inhibition = 100%	

	Essential oils	Disc diffusion method	*A. niger*	MIC = 6.25 *μ*g/mL MBC = 12.50 *μ*g/mL	[11]
Leaves	Hexane	Agar well diffusion techniques	*Saccharomyces cerevisiae*	Ф = 25	[63]
			*Aspergillus niger*	Ф = 26 mm	
Leaves	Petroleum ether	Agar well diffusion techniques	*Saccharomyces cerevisiae*	Ф = 24 mm	[63]
			*Aspergillus niger*	Ф = 27 mm	
Aerial parts	Essential oils	Disc diffusion method	*Candida albicans*	MIC < 3.19 *μ*g/mL	[67]
			*Candida tropicalis*	MIC < 3.19 *μ*g/mL	
Leaves	Ethanol extract		*Fusarium oxysporum* f.sp. *lentis*	Inhibition = 1oo%	[68]
Leaves	Essential oil	Agar diffusion method	*Aspergillus niger* (ATCC 9763)	Ф = 19 mm	[69]
			*Candida albicans* (ATCC 7596)	Ф = 18 mm	

	Essential oil	Agar well diffusion method	*Aspergillus niger*	Ф = 15.7 ± 0.09 mm	[70]
			*Aspergillus* spp.,	Ф = 13 ± 0.13 mm	
			*Candida albicans*	Ф = 11.8 ± 0.10 mm	
			*Rhizopus nigricans*	No inhibition	

Leaves	Essential oil	Agar diffusion method	*Candida albicans*	Ф = 16 mm at concentration of 100 mg/mL	[71]
Leaves	Essential oil	Agar well diffusion method	*Mucor ramamnianus* (ATCC 9314)	Ф = 40 mm	[72]
			*Aspergillus ochraceus* (NRRL 3174)	Ф = 43 mm	
			*Candida albicans* (IPA 200)	Ф = 21 mm	
			*Saccharomyces cerevisiae* (ATCC 4226 A)	Ф = 25 mm	

**Table 4:** Antibacterial activity of *Mentha spicata*.

Parts used	Extracts	Methods used	Strains tested	Key results	References
Leaves	Essential oils	Broth microdilution method	*Staphylococcus aureus* (ATCC 14458)	MIC = 3.2 *μ*L/mL	[6]
			*Staphylococcus epidermidis* (ATCC 12228)	MIC = 1.6 *μ*L/mL	
			*Bacillus cereus* (ATCC 11778)	MIC = 1.6 *μ*L/mL	
			*Listeria monocytogenes* (ATCC 7644)	MIC = 3.2 *μ*L/mL	
			*Escherichia coli* (ATCC 11229)	MIC = 3.2 *μ*L/mL	
			*Salmonella enterica* subsp. *enterica serovar typhimurium* (ATCC 13311)	MIC = 1.6 *μ*L/mL	
			*Salmonella. enterica* subsp. *enterica serovar typhi* (ATCC 19214)	MIC = 1.6 *μ*L/mL	
			*Shigella flexneri* (ATCC 12022)	MIC = 3.2 *μ*L/mL	

Aerial parts	Essential oils	Disc diffusion assay	*P. aeruginosa* (ATCC 27853)	No inhibition	[7]
			*Escherichia coli (*ATCC 25922)	Ф = 9 mm	
			*Staphylococcus aureus* (ATCC 25923)	Ф = 11 mm	
			*Staphylococcus epidermidis*	Ф = 10 mm	
			*Streptococcus pneumoniae*	Ф = 13 mm	
			*Streptococcus pyogenes*	Ф = 16 mm	
			*Klebsiella pneumoniae*	Ф = 8 mm	
			*Salmonella typhi*	Ф = 8 mm	
			*Shigella sonnei*	Ф = 9 mm	

Leaves	Ethanol extract	Disc diffusion assay	*Salmonella paratyphi*	Ф = 17.00 ± 2.00 mm	[74]
			*Shigella boydii*	Ф = 31.67 ± 1.53 mm	
			*Staphylococcus aureus*	Ф = 23.00 ± 1.00 mm	
			*Escherichia coli*	Ф = 9.00 ± 1.00 mm	
			*Vibrio cholerae*	Ф = 12.00 ± 1.00 mm	
			*Pseudomonas aeruginosa*	Trace activity	
			*Enterococcus faecalis*	No activity	
			*Salmonella typhi*	Trace activity	
			*Proteus vulgaris*	No activity	
			*Klebsiella pneumoniae*	No activity	

Leaves	Hexane fraction	Disc diffusion assay	*Salmonella paratyphi*	Ф = 25.67 ± 2.08 mm	[74]
			*Shigella boydii*	Ф = 36.00 ± 1.00 mm	
			*Staphylococcus aureus*	Ф = 22.33 ± 1.53 mm	
			*Escherichia coli*	Ф = 10.67 ± 2.52 mm	
			*Vibrio cholerae*	Ф = 18.67 ± 0.58 mm	
			*Pseudomonas aeruginosa*	Trace activity	
			*Enterococcus faecalis*	No activity	
			*Salmonella typhi*	No activity	
			*Proteus vulgaris*	No activity	
			*Klebsiella pneumoniae*	No activity	

Leaves	Chloroform	Disc diffusion assay	*Salmonella paratyphi*	Ф = 22.67 ± 2.52 mm	[74]
			*Shigella boydii*	Ф = 34.00 ± 1.00 mm	
			*Staphylococcus aureus*	Ф = 24.00 ± 1.00 mm	
			*Escherichia coli*	Ф = 18.67 ± 1.53 mm	
			*Vibrio cholerae*	Ф = 16.00 ± 1.00 mm	
			*Pseudomonas aeruginosa*	Ф = 12.33 ± 1.53 mm	
			*Enterococcus faecalis*	Ф = 8.33 ± 0.58 mm	
			*Salmonella typhi*	No activity	
			*Proteus vulgaris*	No activity	
			*Klebsiella pneumoniae*	No activity	

Leaves	Ethyl acetate fraction	Disc diffusion assay	*Salmonella paratyphi*	Ф = 20.67 ± 1.53 mm	[74]
			*Shigella boydii*	Ф = 32.67 ± 2.52 mm	
			*Staphylococcus aureus*	Ф = 25.33 ± 0.58 mm	
			*Escherichia coli*	Ф = 18.33 ± 1.53 mm	
			*Vibrio cholerae*	Ф = 17.33 ± 1.53 mm	
			*Pseudomonas aeruginosa*	Ф = 8.00 ± 1.00 mm	
			*Enterococcus faecalis*	No activity	
			*Salmonella typhi*	No activity	
			*Proteus vulgaris*	No activity	
			*Klebsiella pneumoniae*	No activity	

Leaves	Aqueous fraction	Disc diffusion assay	*Salmonella paratyphi*	Ф = 22.33 ± 2.52 mm	[74]
			*Shigella boydii*	Ф = 36.00 ± 1.00 mm	
			*Staphylococcus aureus*	Ф = 31.00 ± 1.00 mm	
			*Escherichia coli*	Ф = 21.00 ± 1.00 mm	
			*Vibrio cholerae*	Ф = 20.33 ± 0.58 mm	
			*Pseudomonas aeruginosa*	Ф = 10.00 ± 1.00 mm	
			*Enterococcus faecalis*	No activity	
			*Salmonella typhi*	Trace activity	
			*Proteus vulgaris*	No activity	
			*Klebsiella pneumoniae*	No activity	

Aerial parts	Essential oil	Microdilution method	*Staphylococcus epidermidis*	MIC = 32 *μ*g/mL	[48]
			*Escherichia coli*	MIC = 64 *μ*g/mL	

Aerial parts	Volatile oil	Disk diffusion method	*Xanthomonas* spp. ZI378	Ф = 14 mm	[64]
			*Xanthomonas* spp. ZI376	Ф = 14 mm	
			*Xanthomonas* spp. ZI375	Ф = 13 mm	
			*Xanthomonas* spp. ZI373	Ф = 13 mm	
			*Xanthomonas* spp. ZI370	Ф = 13 mm	
			*Xanthomonas* spp. ZI368	Ф = 13 mm	
			*Xanthomonas* spp. ZI366	Ф = 12 mm	
			*Xanthomonas* spp. ZI365	Ф = 16 mm	

Leaves	Essential oil	Agar disc diffusion method Microbroth dilution	*Staphylococcus aureus* (ATCC 29213)	Ф = 19 ± 1.73 mm	[75]
				MIC = 1.25 *μ*g/mL	
				MBC = 1.25 *μ*g/mL	
			*Escherichia coli* (ATCC 25922)	Ф = 13.66 ± 1.1 mm	
				MIC = 1.25 *μ*g/mL	
				MBC = 2.5 *μ*g/mL	
			*Pseudomonas aeruginosa* (ATCC 27853)	Ф = 9.5 ± 0.70 mm	
				MIC > 10	
				MBC > 10	

Leaves	Essential oil	Disc method	MRSA (ATCC 43300)	Ф = 24.0 ± 1.0 mm	[65]
			*Bacillus subtilis* (ATCC 6633)	Ф = 17.7 ± 0.6 mm	
			*Staphylococcus aureus* (NCC B9163)	Ф = 14.3 ± 1.5 mm	
			*Escherichia coli* (ATCC 25922)	Ф = 11.0 ± 1.0 mm	
			*Pseudomonas aeruginosa* (ATCC 27853)	Ф = 6.0 ± 0.0 mm	
			*Klebsiella pneumonia* E47	Ф = 10.3 ± 0.6 mm	

Leaves	Essential oil	Spots method	MRSA (ATCC 43300)	Ф = 22.3 ± 1.5 mm	[65]
			*Bacillus subtilis* (ATCC 6633)	Ф = 32.7 ± 0.6 mm	
			*Staphylococcus aureus* (NCC B9163)	Ф = 20.3 ± 0.6 mm	
			*Escherichia coli* (ATCC 25922)	Ф = 22.0 ± 1.0 mm	
			*Pseudomonas aeruginosa* (ATCC 27853)	Ф = 6.0 ± 0.0 mm	
			*Klebsiella pneumonia* (E47)	Ф = 17.3 ± 0.6 mm	

Aerial parts	Essential oil	Disc diffusion method	*Escherichia coli*	Ф = 14 ± 0.6 mm	[76]
			*Salmonella enterica* subsp. *enterica*	Ф = 10 ± 0.8 mm	
			*Pasteurella multocida*	Ф = 12 ± 1.0 mm	
			*Staphylococcus aureus*	Ф = 9 ± 1.1 mm	

	Essential oils	Disc diffusion assay Microwell dilution assay	*Escherichia coli* (O157H7)	Ф = 10 mm	[77]
				MIC = 2.26 ± 0.11 *μ*g/mL	
				MBC = 3.66 ± 0.11 *μ*g/mL	
			*Listeria monocytogenes*	Ф = 11 mm	
				MIC = 1.33 ± 0.11 *μ*g/mL	
				MBC = not observed	

Leaves	Essential oils	Disc diffusion technique Checker board technique	*Staphylococcus aureus* 29737	Ф = 10.0 mm	[78]
				MIC = 10 *μ*g/mL	
			*Staphylococcus aureus* ML 267	Ф = 10.0 mm	
				MIC = 10 *μ*g/mL	
			*Suillus luteus* 9341	Ф = 11.0 mm	
				MIC = 5 *μ*g/mL	
			*Bacillus pumilus* 8241	Ф = 10.0 mm	
				MIC = 10 *μ*g/mL	
			*Bacillus subtilis* (ATCC)	Ф = 11.0 mm	
				MIC = 10 *μ*g/mL	
			*Escherichia coli* (ATCC 10536)	Ф = 9.0 mm	
				MIC = 50 *μ*g/mL	
			*Escherichia coli* VC Sonawave 3 : 37 C	Ф = 9.0 mm	
				MIC = 50 *μ*g/mL	
			*Escherichia coli* (CD/99/1)	Ф = 9.5 mm	
				MIC = 50 *μ*g/mL	
			*Escherichia coli* (RP4)	Ф = 9.0 mm	
				MIC = 25 *μ*g/mL	
			*Escherichia coli* (18/9)	Ф = 9.0 mm	
				MIC = 25 *μ*g/mL	
			*Escherichia coli* (K88)	Ф = 8.5 mm	
				MIC = 25 *μ*g/mL	
			*Shigella dysenteriae* L.	Ф = 10.0 mm	
				MIC = 10 *μ*g/mL	
			*Shigella sonnei* 1	Ф = 10.0 mm	
				MIC = 10 *μ*g/mL	
			*Shigella sonnei* BCH 217	Ф = 12.0 mm	
				MIC = 5 *μ*g/mL	
			*Shigella flexneri* type 6	Ф = 9.0 mm	
				MIC = 10 *μ*g/mL	
			*Shigella boydii* 937	Ф = 9.5 mm	
				MIC = 10 *μ*g/mL	
			*Pseudomonas aeruginosa* (ATCC 25619)	Ф = 11.0 mm	
				MIC = 10 *μ*g/mL	
			*Vibrio cholerae* 2	Ф = 10.0 mm	
				MIC = 50 *μ*g/mL	
			*Vibrio cholerae* 785	Ф = 10.0 mm	
				MIC = 50 *μ*g/mL	
			*Vibrio cholerae* 1037	Ф = 9.0 mm	
				MIC = 50 *μ*g/mL	

Leaves	Essential oils	Agar well diffusion method Dilution method	*Staphylococcus aureus*	Ф = 17 ± 0.01 mm	[79]
				MIC = 0.4 ± 0.01 *μ*g/mL	
			*Escherichia coli*	Ф = 14 ± 0.01 mm	
				MIC = 0.5 ± 0.02 *μ*g/mL	
			*Erwinia carotovora*	Ф = 14 ± 0.01 mm	
				MIC = 0.5 ± 0.02 *μ*g/mL	
			*Bacillus subtilis*	Ф = 17 ± 0.01 mm	
				MIC = 0.6 ± 0.01 *μ*g/mL	
			*Xanthomonas campestris*	Ф = 22 ± 0.01 mm	
				MIC = 0.5 ± 0.02 *μ*g/mL	
			*Klebsiella pneumoniae*	Ф = 20 ± 0.01 mm	
				MIC = 0.4 ± 0.01 *μ*g/mL	
Leaves	Essential oils	Diffusion method	*Bacillus subtilis*	Ф = 11.5 ± 0.61 mm	[80]
			*Staphylococcus aureus*	Ф = 13 ± 1.52 mm	
			*Staphylococcus epidermidis*	Ф = 11.2 ± 1.61 mm	
			*Escherichia coli*	Ф = 21 ± 0.90 mm	
			*Pseudomonas aeruginosa*	Ф = 16 ± 1.9 mm	
			*Salmonella enterica* subsp.	Ф = 18 ± 1.33 mm	

Aerial parts	Essential oils	Disc diffusion method Microdilution broth assay	*Staphylococcus aureus*	Ф = 26.0 ± 1.1 mm	[5]
				MIC = 0.07 ± 0.00 *μ*g/mL	
			*Bacillus subtilis*	Ф = 27.1 ± 1.1 mm	
				MIC = 0.05 ± 0.00 *μ*g/mL	
			*Pasteurella multocida*	Ф = 24.3 ± 0.9 mm	
				MIC = 0.12 ± 0.01 *μ*g/mL	
			*Escherichia coli*	Ф = 20.3 ± 0.9 mm	
				MIC = 0.21 ± 0.01 *μ*g/mL	

Whole plant	Essential oils	Disc diffusion method	*Escherichia coli*	MIC = 1/250 (V/V)	[81]
				MBC = 1/250 (V/V)	

Not reported	Essential oil	Disc diffusion method	*Escherichia coli*	MIC = 1.56 *μ*g/mL	[11]
				MBC = 25 *μ*g/mL	
			*Staphylococcus aureus*	MIC = 25 *μ*g/mL	
				MBC = 50 *μ*g/mL	
			*Saccharomyces cerevisiae*	MIC = 0.78 *μ*g/mL	
				MBC = 6.25 *μ*g/mL	
			*Penicillium citrinum*	MIC = 3.12 *μ*g/mL	
				MBC = 12.50 *μ*g/mL	

Leaves	Hexane	Agar well diffusion techniques	*Pseudomonas aeruginosa*	Ф = 15 mm	[63]
			*Bacillus subtilis*	Ф = 10 mm	
			*Escherichia coli*	Ф = 25 mm	
			*Staphylococcus aureus*	Ф = 26 mm	

Leaves	Petroleum ether	Agar well diffusion techniques	*Pseudomonas aeruginosa*	Ф = 17 mm	[63]
			*Bacillus subtilis*	Ф = 12 mm	
			*Escherichia coli*	Ф = 26 mm	
			*Staphylococcus aureus*	Ф = 27 mm	

Aerial parts	Essential oils	Disc diffusion method	*Staphylococcus aureus*	MIC = 15.6 *μ*g/mL	[67]
			*Enterococcus faecalis*	MIC = 125 *μ*g/mL	
			*Pseudomonas aeruginosa*	MIC = 125 *μ*g/mL	
			*Escherichia coli*	MIC < 3.19 *μ*g/mL	

Leaves	Essential oil	Broth microdilution method	*Serratia* spp.	MIC = 4.75 mg/mL	[82]
				MBC > 9.5 mg/mL	
			*Salmonella* spp.	MIC = 2.37 mg/mL	
				MBC > 9.5 mg/mL	
			*Kluyvera* spp.	MIC = 2.37 mg/mL	
				MBC > 9.5 mg/mL	
			*Klebsiella* spp.	MIC = 2.37 mg/mL	
				MBC = 9.5 mg/mL	
			*Escherichia coli* (F5)	MIC = 2.37 mg/mL	
				MBC > 9.5 mg/mL	
			*Escherichia coli* (F17)	MIC > 9.5 mg/mL	
				MBC > 9.5 mg/mL	
			*Escherichia coli* (CS31 A)	MIC = 2.37 mg/mL	
				MBC = 9.5 mg/mL	

Aerial parts	Essential oil	Disc diffusion method	MRSA	Ф = 17.5 ± 0.7 mm	[31]
			*Staphylococcus aureus* (ATCC 6538)	Ф = 11 ± 1.4 mm	
			*Pseudomonas aeruginosa*	Ф = 21 ± 8.4 mm	
			*Escherichia coli* Q157 : H7	Ф = 20.5 ± 2.1 mm	
			*Bacillus cereus* (CCM99)	Ф = 22.5 ± 0.7 mm	
			*Enterococcus faecium* (DSM 13590)	Ф = 13 ± 4.2 mm	

Leaves	Essential oil	Broth microdilution method	*Staphylococcus aureus* (ATCC 6538)	MIC = 10 *μ*g/mL	[83]
				MBC = 10 *μ*g/mL	
			*Staphylococcus aureus* (ATCC 29213)	MIC = 8 *μ*g/mL	
				MBC = 8 *μ*g/mL	
			*Bacillus subtilis* (ATCC 6633)	MIC = 2.5 *μ*g/mL	
				MBC = 5 *μ*g/mL	
			*Bacillus cereus* (ATCC 11774)	MIC = 2.5 *μ*g/mL	
				MBC = 5 *μ*g/mL	
			*Listeria monocytogenes* (ATCC 19118)	MIC = 2.5 *μ*g/mL	
				MBC = 2.5 *μ*g/mL	
			*Salmonella typhimurium* (ATCC 14028)	MIC = 10 *μ*g/mL	
				MBC = 10 *μ*g/mL	
			*Escherichia coli* O157 : H7 (ATCC 10536)	MIC = 10 *μ*g/mL	
				MBC = 10 *μ*g/mL	

Not reported	Essential oil	Microdilution method	*Staphylococcus aureus*	MIC = 0.005 *μ*g/mL	[84]
			*Bacillus subtilis*	MIC = 0.005 *μ*g/mL	
			*Bacillus cereus*	MIC = 0.005 *μ*g/mL	
			*Listeria monocytogenes*	MIC = 0.005 *μ*g/mL	
			*Salmonella typhimurium*	MIC = 0.005 *μ*g/mL	
			*Escherichia coli* O157 : H7	MIC = 0.005 *μ*g/mL	

Leaves	Essential oil	Agar diffusion method	*Escherichia coli* (ATCC 25922)	Ф = 17 mm	[69]
			*Bacillus subtilis* (NCTC 8236)	Ф = 16 mm	

Not reported	Decanted essential oil	Disc diffusion assay	*Staphylococcus epidermidis*	Ф = 2 mm	[85]
			*Enterococcus faecalis*	Ф = 5 mm	
			*Streptococcus mutans*	Ф = 5 mm	
			*Escherichia coli*	Ф = 6 mm	
			*Pseudomonas aeruginosa*	No inhibition	

Not reported	Recovered essential oil	Disc diffusion assay	*Staphylococcus epidermidis*	Ф = 2 mm	[85]
			*Enterococcus faecalis*	Ф = 4 mm	
			*Streptococcus mutans*	Ф = 5 mm	
			*Escherichia coli*	Ф = 6 mm	
			*Pseudomonas aeruginosa*	No inhibition	

Not reported	Essential oil	Agar well diffusion method	*Escherichia coli*	Ф = 14 ± 0.05 mm	[70]
			*Salmonella typhi*	No inhibition	
			*Salmonella paratyphi*	No inhibition	
			*Staphylococcus aureus*	Ф = 21 ± 0.09 mm	
			*Klebsiella pneumoniae*	Ф = 12.7 ± 0.07 mm	
			*Pseudomonas aeruginosa*	No inhibition	
			*Acinetobacter* spp.	Ф = 18 ± 0.11 mm	

Leaves	Essential oil	Agar diffusion method	*Bacillus subtilis*	Ф = 15 mm at a concentration of 100 mg/mL	[71]
			*Escherichia coli*	Ф = 17 mm at concentration of 100 mg/mL	
			*Staphylococcus aureus*	Ф = 16 mm at a concentration of 100 mg/mL	
			*Pseudomonas aeruginosa*	Ф = 16 mm at a concentration of 100 mg/mL	

Aerial parts	Essential oil	Agar diffusion method	*Pseudomonas aeruginosa* (ATCC 27853)	No inhibition	[7]
			*Escherichia coli* (ATCC 25922)	Ф = 9 mm	
			*Staphylococcus aureus* (ATCC 25923)	Ф = 11 mm	
			*Staphylococcus epidermidis*	Ф = 10 mm	
			*Streptococcus pneumoniae*	Ф = 13 mm	
			*Streptococcus pyogenes*	Ф = 16 mm	
			*Klebsiella pneumoniae*	Ф = 8 mm	
			*Salmonella typhi*	Ф = 8 mm	
			*Shigella sonnei*	Ф = 9 mm	

Leaves	Essential oil	Agar-well diffusion assay Broth microdilution assay	*Staphylococcus aureus*	Ф = 32.00 ± 2.65 mm	[86]
				MIC = 0.25% (v/v)	
				MBC = 0.25% (v/v)	
			*Pseudomonas aeruginosa*	Ф = 13.33 ± 1.53 mm	
				MIC = 0.5% (v/v)	
				MBC = 2% (v/v)	
			*Listeria monocytogenes*	Ф = 26.67 ± 2.08 mm	
				MIC = 0.25% (v/v)	
				MBC = 0.25% (v/v)	
			*Bacillus subtilis*	Ф = 17.00 ± 2.00 mm	
				MIC = 1% (v/v)	
				MBC = 1% (v/v)	
			*Proteus mirabilis*	Ф = 29.33 ± 1.53 mm	
				MIC = 0.5% (v/v)	
				MBC = 1% (v/v)	
			*Escherichia coli*	Ф = 15.33 ± 1.89 mm	
				MIC = 2% (v/v)	
				MBC > 2% (v/v)	

Whole plant			*Staphylococcus aureus* (MBLA)	Ф = 18 ± 1.34 mm	[87]
				MIC = 4% (v/v)	
				MBC ˃ 8% (v/v)	
			*Staphylococcus aureus* 976	Ф = 9 ± 1.9 mm	
			*Listeria monocytogenes*	Ф = 21 ± 3.11 mm	
				MIC = 1% (v/v)	
				MBC = 4% (v/v)	
			*Staphylococcus aureus* 994	Ф = 7 ± 0.66 mm	
			*Bacillus subtilis* 6633	Ф = 15 ± 0.80 mm	
			*Escherichia coli* K12	Ф = 9 ± 0.65 mm	
			*Pseudomonas aeruginosa* IH	No activity	
			*Proteus mirabilis*	Ф = 19 ± 0.41 mm	
				MIC = 4% (v/v)	
				MBC ˃ 8% (v/v)	

Leaves	Essential oil	Agar well diffusion method	*Klebsiella pneumoniae* (CIP8291)	Ф = 25 mm	[72]
			*Escherichia coli* (ATCC10536)	No activity	
			*Staphylococcus aureus* (CIP7625)	No activity	
			*Listeria monocytogenes* (Scott A 724)	Ф = 29 mm	

Leaves	Essential oil	Agar well diffusion method	*Escherichia coli*	Ф = 8 mm at a concentration of 500 *μ*L/mL	[88]
			*Salmonella choleraesuis*	Ф = 13 mm at a concentration of 500 *μ*L/mL	
			*Staphylococcus aureus*	Ф = 11 mm at a concentration of 500 *μ*L/mL	
			*Listeria monocytogenes*	Ф = 9.5 mm at a concentration of 500 *μ*L/mL	

Aerial parts	Essential oil	Disc diffusion method	*Escherichia coli*	MIC = 2.5* μ*L/mL	[89]
				MBC = 2.5* μ*L/mL	
			*Streptococcus D*	MIC = 2.5* μ*L/mL	
				MBC = 2.5* μ*L/mL	
			*E. faecalis*	MIC = 2.5* μ*L/mL	
				MBC = 2.5* μ*L/mL	
			*K. pneumoniae*	MIC = 2.5* μ*L/mL	
				MBC = 2.5* μ*L/mL	

**Table 5:** Antiparasitic activity of *Mentha spicata*.

Part used	Extracts	Tested strains	Key results	Reference
Leaves	Dried plant	*Varroa destructor*	Killed 26.20% of *Varroa*	[90]
			Infestation rates = 13.18%	
			Reduced the infestation rate of 2.35%	
			Mortality rate = 30.65%	

Leaves	Essential oils	*Tetranychus turkestani*	LC_50_ = 15.3 mL/L	[91]
			LC_95_ = 23.4 mL/L	
			Mortality = 100% at concentration of 20 *μ*L/L	

**Table 6:** Insecticidal activity of *Mentha spicata*.

Part used	Extracts	Tested strains	Key results	Reference
Leaves	Essential oil	*Rhyzopertha dominica*	LD_50_ = 6.1 *μ*L/mL	[65]
			Mortality = 43% after 96 hours at a concentration of 2 *μ*L/mL	
			Repellent effect = 56.2% at a concentration of 2 *μ*L/mL	

Leaves	Essential oil	Rice weevil *Sitophilus oryzae*	LC_50_ = 100.16 *μ*L/L air; LC_95_ = 192.197 *μ*L/L air; mortality = 22% at a concentration of 71.43 *μ*L/L air; LT_50_ = 45.52 h	[93]
Aerial parts	Essential oil	*Callosobruchus chinensis*	Mortality = 100% after 12 h at concentration of 0.1 *μ*L/mL air	[66]
			LC_50_ = 0.003 *μ*L/mL air	
			LC_90_ = 0.005 *μ*L/mL air	
			Repellency value = 100% at 0.025 *µ*L/mL air of oil concentration	
			98% oviposition deterrence at 0.1 *μ*L/mL concentration	

	Essential oil	*Sitophilus granarius*	Mortality = 43% at the 24 h exposure test	[94]
			Mortality = 80% at the 48 h exposure test	

Whole flowering plants	Essential oil	*Acanthoscelides obtectus*	LC_50_ = 1.2 mL/L air, for males	[95]
			LC_50_ = 4.4 mL/L air, for females	
	Essential oil	*Boophilus annulatus*	Embryonated eggs (LC_50_ = 1.20%); unfed larvae (LC_50_ = 0.90%); fed females (LC_50_ = 10.57%)	[96]
Leaves	oil	*Callosobruchus maculatus*	LC_50_ = 235 ppm	[92]
	Essential oil	*Culex quinquefasciatus* Say	LC_50_ = 92 mg/L	[3]
			LC_90_ = 160 mg/L	

Leaves	Essential oil	*C. quinquefasciatus*	LC_50_ = 62.62 ppm; LC_90_ = 118.70 ppm	[38]
			Mortality = 96.8 ± 1.2% at a concentration of 125 ppm	
		*A. aegypti*	LC_50_ = 56.08 ppm; LC_90_ = 110.28 ppm	
			Mortality = 98.1 ± 1.2% at a concentration of 125 ppm	
		*A. stephensi*	LC_50_ = 49.71 ppm; LC_90_ = 100.99 ppm	
			Mortality = 99.6 ± 1.6% at a concentration of 125 ppm	

**Table 7:** Anti-inflammatory activity of *Mentha spicata*.

Used part	Extracts	Experimental approach	Key results	References
Whole plant	Methanol extract	Carrageen-induced paw edema method	Significant dose-dependent reduction of paw edema	[97]
Leaves	Hexane extract	Carrageenan-induced paw edema in rats	Reduced the inflammation with less effectiveness	[98]
			Reduced the inflammation by 0–20%	
	Ethyl acetate extract		Reduced the inflammation by 9–85%	
	Chloroform fraction		The inflammation did not decrease	
	Aqueous fraction		Enhances inflammation by about 7–11%	
	Hexane extract	Cotton pellet-induced granuloma in rats	Reduced inflammation with 20%	
	Ethyl acetate extract		Reduced inflammation with 65%	
	Chloroform fraction		Reduced inflammation with 20%	
	Aqueous fraction		Reduced inflammation with 54%	

Leaves	Methanol extract	Irinotecan-induced mucositis in mice	Significantly decreased both jejunal tissue IL-1*β* and fecal *β*-glucuronidase activity	[99]
			Improvements in mucositis features	

**Table 8:** Antidiabetic effects of *Mentha spicata*.

Part used	Extracts	Dose	Model	Keys results	References
Leaves	Aqueous ethanolic extract	200 mg/kg and 400 mg/kg bodyweight	Alloxan-induced hyperglycemic rats	Reduced blood glucose level, reduced serum cholesterol, triglycerides, LDL, and VLDL and increased bodyweights and HDL levels	[101]
Leaves	Phenolic extract	200 mg/kg bodyweight	Alloxan-induced hyperglycemic rats	Significant decrease in glucose concentration of blood serum; significant decrease in cholesterol and TG; significant increase in plasma HDL; significant decrease in plasma LDL, VLDL	[100]
Leaves	Aqueous extract	300 mg/kg bodyweight	Alloxan-induced hyperglycemic rats	Decreased blood glucose level; decreased bodyweight; significant reduction of total cholesterol, triglyceride, and LDL-cholesterol levels; significant increase in plasma HDL; significant reduction in the level of MDA	[13]
Roots	Butanol extract	100 mg/kg bodyweight	Streptozotocin-induced hyperglycemic rats	Increased bodyweight; reduced blood glucose	[8]
Leaves	Essential oil	200 *μ*L	*α*-Glucosidase inhibitory assay	IC_50_ = 86.93 ± 2.43 *μ*g/mL	[86]
		250 *μ*L	*α*-Amylase inhibitory assay	IC_50_ = 101.72 ± 1.86 *μ*g/mL	

**Table 9:** Antioxidant activity of *Mentha spicata*.

Parts used	Extracts	Methods used	Key results	References
Whole plant	Methanol extract	DPPH	IC_50_ = 65.13 ± 1.29 *μ*g/mL	[103]
		ABTS	IC_50_ = 52.31 ± 0.81 *μ*g/mL	
Aerial parts	Ethanol extract	DPPH	IC_50_ = 87.89 *μ*g/mL	[102]
		ABTS	IC_50_ = 173.80 *μ*g/mL	
Aerial parts	Essential oil	DPPH	IC_50_ = 3450 ± 172.5 *μ*g/mL	[48]
		ABTS	IC_50_ = 40.2 ± 0.2 *μ*g/mL	
		FRAP	IC_50_ = 215 ± 4.50 *μ*g/mL	
Leaves	Essential oil	DPPH	IC_50_ = 9544.6 ± 196.2 *μ*g/mL	[65]
		ABTS	IC_50_ = 36.2 ± 3.2 *μ*g/mL	
		Reducing power	RP_50_ = 452.3 ± 0.4 *μ*g/mL	
		Phosphomolybdate	RP_50_ = 53.3 ± 2.8 *μ*g/mL	
Leaves	Essential oil	DPPH	IC_50_ = 10 ± 0.24 *μ*g/mL	[104]
		Superoxide anion	IC_50_ = 1.33 ± 0.10 *μ*g/mL	
Aerial parts	Ethanol-water extract	DPPH	IC_50_ = 105.8 ± 3.98 *μ*g/mL	[9]
		Nitric oxide radical scavenger	IC_50_ = 210.6 ± 7.7 *μ*g/mL	
		Metal chelating	IC_50_ = 757.4 ± 29.5 *μ*g/mL	
		Scavenging of H_2_O_2_	IC_50_ = 631.1 ± 26.0 *μ*g/mL	
Seeds	Methanol extract	DPPH	Inhibition = 89.91 ± 2.12%	[33]
Leaves	Ethanol extract	DPPH	IC_50_ = 16.2 ± 0.2 *μ*g/mL	[105]
		ABTS	IC_50_ = 10.3 ± 0.9 *μ*g/mL	
		TEAC	TEAC = 0.90 ± 0.07 mM	
Leaves	Essential oils	DPPH	IC_50_ = 8.81 *μ*L/mL	[78]
Leaves	Essential oils	DPPH	IC_50_ = 41,23 *μ*L/mL	[80]
Aerial parts	Essential oils	DPPH	IC_50_ = 13.3 ± 0.6 *μ*L/mL	[5]
	Essential oil	DPPH	IC_50_ = 72.07 ± 0.34 mg/mL	[11]
Aerial parts	Water extract	DPPH	Inhibition = 74.2 ± 0.2%	[106]
		*β*-Carotene	Inhibition = 79.1 ± 2.4%	
Leaves	Water extract	DPPH	EC_50_ = 336 ± 3 *μ*g/mL	[107]
		Reducing power	EC_50_ = 198 ± 2 *μ*g/mL	
		TBARS	EC_50_ = 152 ± 5 *μ*g/mL	
Leaves	Essential oil	DPPH	IC_50_ = 21.19 ± 7.17 *μ*g/mL	[82]
		Reducing power	IC_50_ = 2.28 ± 0.68 *μ*g/mL	
		DPPH	Inhibition = 30.52 ± 0.09% at a concentration of 10 *μ*g/mL	
Aerial parts	Essential oil	DPPH	IC_50_ = 3.08 ± 0.07 *μ*g/mL	[108]
		Reducing power	EC_50_ = 2.49 ± 0.07 *μ*g/mL	
		Chelating power	IC_50_ = 6.33 ± 0.12 *μ*g/mL	
		*β*-Carotene	IC_50_ = 6.4 ± 0.07 *μ*g/mL	
Leaves	Essential oil	*β*-Carotene-linoleic acid	Antioxidant activity = 25.37% at 500 *μ*g/mL	[44]
		DPPH	Antioxidant activity = 13.81% at 500 *μ*g/mL	
Aerial parts	Ethanol extract	DPPH	Radical scavenging effect = 18.34 ± 2.2% at a concentration of 0.4 mg/mL	[50]
	Essential oil	DPPH	IC_50_ ˃ 2.5 *μ*g/mL	[87]
Leaves	Essential oil	DPPH	IC_50_ = 80.45 ± 1.86 *μ*g/mL	[86]
		FRAP	IC_50_ = 101.78 ± 3.14 *μ*g/mL	
		ABTS	IC_50_ = 139.59 ± 3.12 *μ*g/mL	
Leaves	Essential oil	ABTS	IC_50_ = 195.1 ± 4.2 mg/L	[72]
		DPPH	IC_50_ = 3476.3 ± 133 mg/L	

**Table 10:** Toxicity study of *Mentha spicata*.

Parts used	Extracts	Experimental approaches	Key results	References
Aerial parts	Methanol extract	The animals treated with a single dose of 5000 mg/kg of *M. spicata* extract by oral gavage	No mortality during the observation period	[113]
			No toxicologically significant hematological and biochemical changes	
			Any morphological changes in the heart, liver, kidney, and lung tissues of the rats	
			LD_50_ is considered to be >5000 mg/kg	
Leaves	Ethanolic extract	The animals treated with a single dose of 10000, 12000, 14000, 16000, and 18000 mg/kg BW of *M. spicata* extract by oral gavage	NOEL dose for *M. spicata* was 10000 mg/kg	[114]
			100% mortality at 18000 mg/kg BW	
			LD_50_ = 13606 mg/kg BW	

Leaves	Ethanolic extract	The animals treated with 500, 1000, and 1500 mg/kg BW daily for 28 days	No signs of toxicity; no mortalities; no significant change in bodyweight; significant increase in WBC, Lym, and MCHC levels; significant reduction in HCT level; significant increase in AST levels; unaffected serum creatinine and urea; no significant histopathological change	[114]
Whole plant	Methanol extract	Mice treated with a single dose of 500, 1000, and 2000 mg/kg of *M. spicata* extract by oral gavage	No mortality during the observation period	[97]
Aerial parts	Essential oil	EO (0.05–0.5 mL) orally administered to mice (*Mus musculus* L., average weight 30.0 g, age 3 months)	LD_50_ = 8342.33 *μ*L/kg	[66]
Leaves	Ethanolic extract	The animals treated with 500, 1000, and 1500 mg/kg BW daily for 28 days	No signs of toxicity; no mortalities; no significant change in bodyweight; significant increase in WBC, Lym, and MCHC levels; significant reduction in HCT level; significant increase in AST levels; unaffected serum creatinine and urea; no significant histopathological change	[114]
Whole plant	Methanol extract	Mice treated with a single dose of 500, 1000, and 2000 mg/kg of *M. spicata* extract by oral gavage	No mortality during the observation period	[97]
Aerial parts	Essential oil	EO (0.05–0.5 mL) orally administered to mice (*Mus musculus* L., average weight 30.0 g, age 3 months)	LD_50_ = 8342.33 *μ*L/kg	[66]

## Data Availability

The data used to support this study are included within the article.

## Supplementary Materials

Supplementary Materials Graphical abstract of this study is attached in supplementary file.
