# Supplementary figures and images for: Medicinal Uses, Phytochemistry, Pharmacology, and Toxicology of Mentha spicata
Source: Evid Based Complement Alternat Med. 2022 Apr 12;2022:7990508. doi: 10.1155/2022/7990508 (PMC9019422; doi:10.1155/2022/7990508)

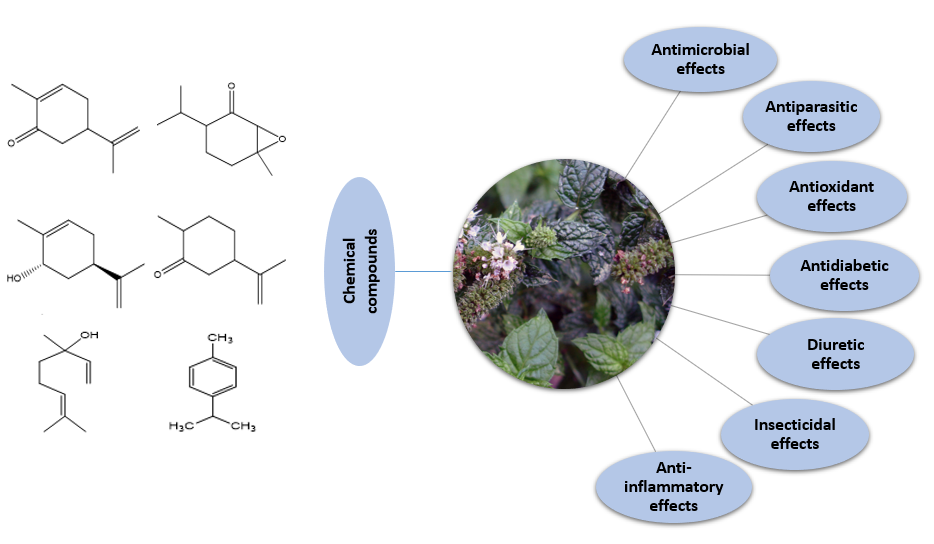

Supplement: Supplementary Materials — Graphical abstract of this study is attached in supplementary file. [file 7990508.f1.docx]
